# Developmental transcriptomics of Chinese cordyceps reveals gene regulatory network and expression profiles of sexual development-related genes

**DOI:** 10.1186/s12864-019-5708-z

**Published:** 2019-05-04

**Authors:** Xiao Li, Fen Wang, Qing Liu, Quanping Li, Zhengming Qian, Xiaoling Zhang, Kuan Li, Wenjia Li, Caihong Dong

**Affiliations:** 1 State Key Laboratory of Mycology, Institute of Microbiology, Chinese Academy of Sciences, NO. 3 Park 1, Beichen West Road, Chaoyang District, Beijing, 100101 China; 2 University of Chinese Academy of Sciences, Beijing, 100039 China; 3 Key Laboratory of State Administration of Traditional Chinese Medicine, Sunshine Lake Pharma Co., LTD, Dongguan, 523850 Guangdong China

**Keywords:** *Ophiocordyceps sinensis*, Weighted gene coexpression network analysis (WGCNA), Hub genes, Mating, Sexual development, Spatiotemporal specificity

## Abstract

**Background:**

Chinese cordyceps, also known as Chinese caterpillar fungus (*Ophiocordyceps sinensis*, syn. *Cordyceps sinensis*), is of particular interest for its cryptic life cycle and economic and ecological importance. The large-scale artificial cultivation was succeeded recently after several decades of efforts and attempts. However, the induction of primordium, sexual development of *O. sinensis* and the molecular basis of its lifestyle still remain cryptic.

**Results:**

The developmental transcriptomes were analyzed for six stages covering the whole developmental process, including hyphae (HY), sclerotium (ST), primordium (PR), young fruiting body (YF), developed fruiting body (DF) and mature fruiting body (MF), with a focus on the expression of sexual development-related genes. Principal component analysis revealed that the gene expression profiles at the stages of primordium formation and fruiting body development are more similar than those of the undifferentiated HY stage. The PR and MF stages grouped together, suggesting that primordium differentiation and sexual maturation have similar expression patterns. Many more DEGs were identified between the ST and HY stages, covering 47.5% of the *O. sinensis* genome, followed by the comparisons between the ST and PR stages. Using pairwise comparisons and weighted gene coexpression network analysis, modules of coexpressed genes and candidate hub genes for each developmental stage were identified. The four mating type loci genes expressed during primordium differentiation and sexual maturation; however, spatiotemporal specificity of gene expression indicated that they also expressed during the anamorphic HY stage. The four mating type genes were not coordinately expressed, suggesting they may have divergent roles. The expression of the four mating type genes was highest in the fertile part and lowest in the sclerotium of the MF stage, indicating that there is tissue specificity. Half of genes related to mating signaling showed as the highest expression in the ST stage, indicating fruiting was initiated in the ST stage.

**Conclusions:**

These results provide a new perspective to understanding of the key pathways and hub genes, and sexual development-related gene profile in the development of Chinese cordyceps. It will be helpful for underlying sexual reproduction, and add new information to existing models of fruiting body development in edible fungi.

**Electronic supplementary material:**

The online version of this article (10.1186/s12864-019-5708-z) contains supplementary material, which is available to authorized users.

## Background

Chinese cordyceps, an entities of Chinese caterpillar fungus (*Ophiocordyceps sinensis*, syn. *Cordyceps sinensis*), parasitize ghost moth larvae and is one of the most valued traditional Chinese medicinal fungi, found exclusively in the Tibetan Plateau [1]. It is well known as ‘Dong Chong Xia Cao’ (winter worm, summer grass) in Chinese, or ‘Hia Tsao Tong Tchong’ in early English translations [2], and “yarsa gumba” in North Sikkim [3]. Chinese cordyceps was commonly used to replenish the kidney and soothe the lung since the Qing dynasty in China. In each edition of the Chinese Pharmacopeia, it has been officially recorded as a drug. It has also been regarded as the Himalayan Viagra [3].

Chinese cordyceps exclusively inhabits the harsh alpine environments of the Qinghai-Tibetan Plateau with an altitude from above 3000 m up to the snow line, including the southwestern regions of China (the Tibetan Autonomous Region and the Qinghai, Sichuan, Yunnan and Gansu provinces) and some countries on the southern slope of Himalayas (Nepal, Bhutan and northeast India) [4]. The price of Chinese cordyceps is extremely high for its medical benefits and dwindling supplies, approximately $20,000 to $40,000 USD per kg, which has led to Chinese cordyceps being regarded as “soft gold” in China. The huge market demand has led to over harvesting, which resulted in it being listed as an ‘endangered species for protection’ in China [5].

The increase in demand and the extremely high price have stimulated interest in the artificial cultivation of the *O. sinensis* fruiting body. The fungus colonizes ghost moth caterpillars (*Hepialus*/*Thitarodes*), forming a parasitic complex that comprises the remains of the caterpillar and the fungal sexual stroma. Due to its complex and long-life cycle, the large-scale artificial cultivation of this fungus has been unsuccessful in China until recently [6]. However, the rigidity of larva after being infected by *O. sinensis* and the induction of fruiting body are still inefficient, resulting in the high cost.

Genome surveys indicated that *O. sinensis* is adapted to extreme cold with putative antifreeze proteins and increased accumulation of lipids and unsaturated fatty acids [7]. Additionally, this fungus displays considerable lineage-specific expansion of gene families functionally enriched for adaptation to low-temperatures, fungal pathogenicity and specialized host infection [8]. At present, the developmental biology of the fruiting body of this fungus is poorly understood. Fresh fruiting bodies of wild *O. sinensis* were sourced from an herb market in Kangding County, Sichuan Province, China and the gene expression in only one developmental stage of this wild sample was analyzed [9]. Recent transcriptome analyses have compared the transcripts of three *O. sinensis* developmental stages (mycelia, sclerotium and stromata) which were also from wild samples; however, the wild *O. sinensis* was divided into only two parts, the sclerotium and stroma [10], which do not represent the different developmental stages. It was reported that the induction of sexual processes may be linked to the cryptic environmental factors specific to the alpine ecosystem of the Tibetan Plateau [7]. Recently, metatranscriptomic analysis of Chinese cordyceps collected from five places on the Qinghai-Tibetan Plateau revealed that the genes involved in sexuality and fruiting-body development were differentially expressed in different samples, implying that the fungus might have been in variable developmental stages on the sampling dates [11].

Sexual identity varies among different fungal species. The mating type (MAT) locus, either MAT1–1 or MAT1–2, controls transitions between heterothallism, homothallism and pseudohomothallism [12, 13]. Zhang et al. [14] revealed for the first time that a MAT1–2-1 existed in tissue and single-ascospore cultures of all examined *O. sinensis*. Genome sequencing analysis found that *O. sinensis* not only possessed MAT1–2-1 but also had MAT1–1 idiomorph among three additional mating-type genes (i.e., MAT1–1-1, MAT1–1-2 and MAT1–1-3) [7]. Additionally, the *O. sinensis* mating-type genes are significantly different from those in closely related fungal pathogens, such as *Beauveri bassiana*, *Cordyceps militaris*, *Metarhizium anisopliae*, *M. acridum* and *Tolypocladium inflatum* [8]. The expression profile of sexual phase-related genes during the development stages of *O. sinensis* will be helpful in understanding the sexual reproduction of this fungus.

Weighted gene coexpression network analysis (WGCNA) has been proven to be an effective method to detect coexpressed modules and hub genes [15]. Based on pairwise correlations, genes with similar expression profiles can be grouped into a model or network by WGCNA, and these models can be correlated to different stages of Chinese cordyceps. First, the gene coexpression network was defined based on the absolute value of the correlation of paired genes. Next, the strength between inter-related genes was defined with adjacency matrix. The correlation between genes and module eigengenes was used to identify hub genes. WGCNA has been used recently for identifying the regulatory networks and hub genes controlling some traits in plants [16, 17] and the key pathways and genes involved in the dynamic changes of hepatocellular carcinoma progression [18].

*O. sinensis* is attractive and there are still many unanswered questions concerning its biology [6, 19]. The fact that this fungus mainly infects and develops under frozen ground over 3000 m high in the wild during its life cycle is the main obstacle for the biological study of this fungus. The success of artificial cultivation provides convenience in sampling during the different developmental stages. In the present study, fungal samples of six developmental stages were harvested from the artificial cultivation workshop and the developmental transcriptomes were analyzed. Using WGCNA, we identified modules of coexpressed genes and candidate hub genes for each developmental stage. In particular, we focused on the differential expression of sexuality-related genes and the expression profiles of four mating genes in the different developmental stages and tissues of *O. sinensis*. This work provides important insights into the molecular networks underlying development and the foundation for improving the artificial cultivation of this fungus.

## Results

### Global transcriptomic analysis

**Fig. 1:**
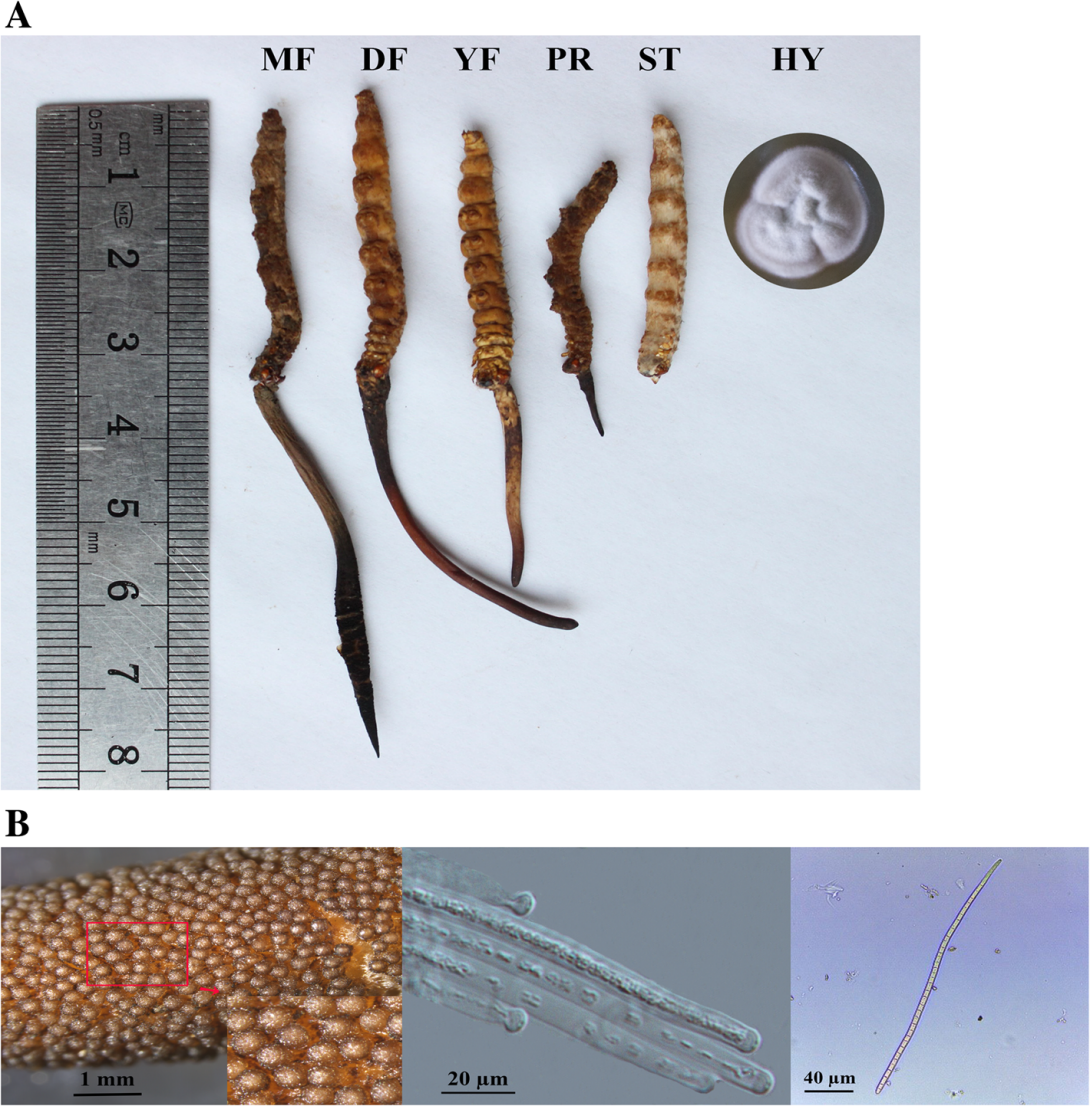
Chinese cordyceps sample collection for RNA sequencing (RNA-Seq). **a**. Different development stages of Chinese cordyceps. HY: the hyphae of Chinese cordyceps; ST: sclerotium (mummified larva) before stroma development; PR: sclerotium with initial stroma (stroma < 1 cm); YF: sclerotium with early stage stroma (1 cm < stroma < 3 cm); DF: sclerotium with developed stroma (stroma > 5 cm); MF: fruiting body with mature perithecia, ascus and ascospores. **b**. Perithecia, young ascospores and mature ascospores of Chinese cordyceps

To describe the patterns of gene expression during the development, 18 libraries were constructed using samples from six growth stages of Chinese cordyceps (Fig. 1a), with three biological replicates for each sample. A total of 550.9 million raw reads were generated by Illumina paired-end sequencing. After cleaning and quality checks, 531.7 million clean reads were obtained, with an average of 29.5 million reads per replicate (Additional file 1: Table S1). More than 85.66% of the reads within each replicate could be mapped to the *O. sinensis* genome [8] (Additional file 1: Table S1). The Q30 percentages for all the sequences (with an error probability of 0.01; a high-quality indicator) in the 18 libraries were over 91%. The Pearson’s correlation coefficient of gene expression between replicates for each sample was more than 0.93 (Additional file 2: Figure S1), indicating good repeatability.

Using an FPKM cutoff value of 0, over 98% of genes were detected and expressed in the 18 samples. The genome-wide distribution of gene transcription levels derived from the RNA-Seq data is described in Additional file 2: Figure S2. On a global scale, all genes could be divided into four categories according to their FPKM values, with the majority of genes moderately expressed (10 ≤ FPKM < 100) in all samples during the development. The ST stage had the greatest number of highly expressed genes (FPKM ≥100), while the HY stage had the fewest. The ST stage had the greatest number of genes expressed at low levels (0 < FPKM ≤10) (Additional file 2: Figure S2). In total, approximately 99.27, 98.77, 98.69, 98.35, 98.52 and 98.60% of genes were expressed in the HY, ST, PR, YF, DF and MF stages, respectively.

### Clustering of gene expression profiles across the six growth stages

**Fig. 2:**
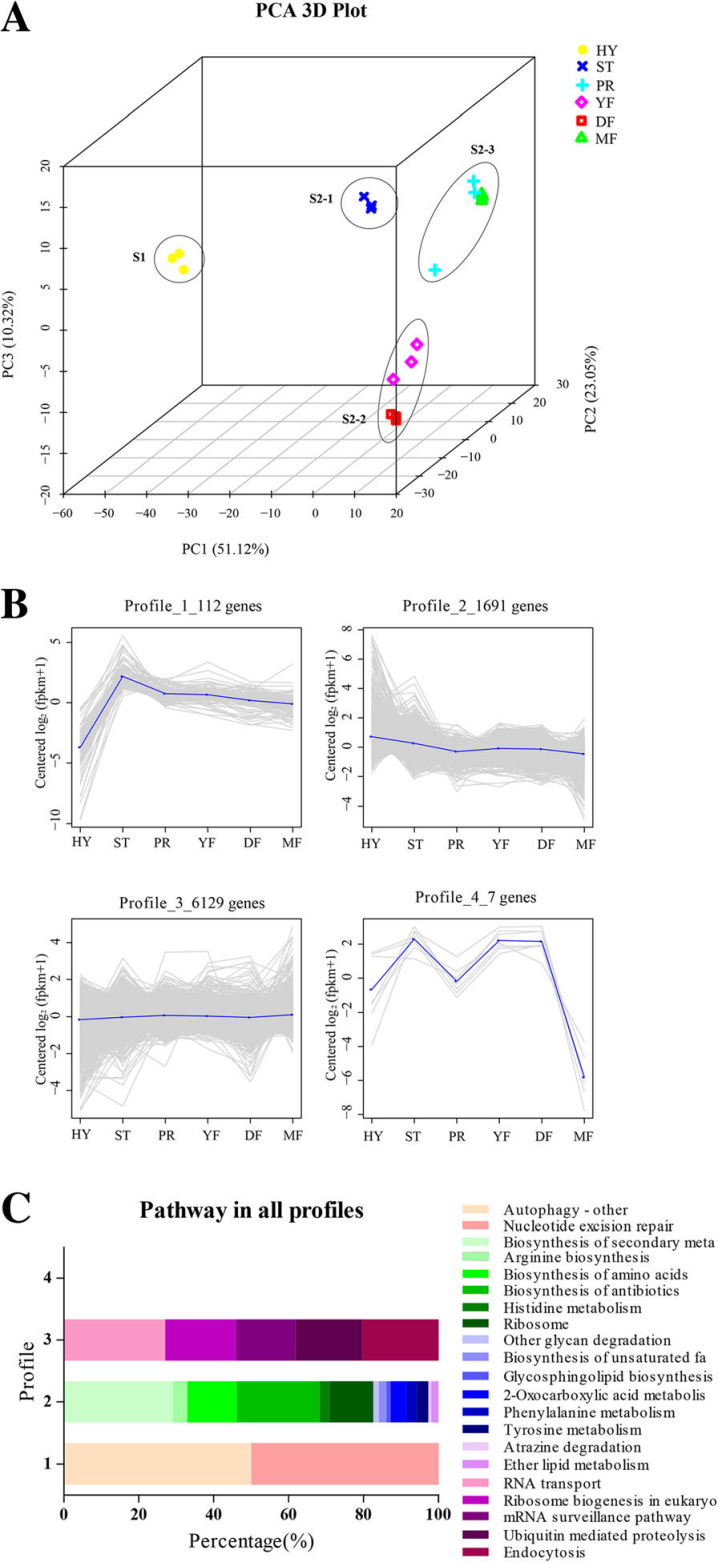
Clustering of gene expression profile across six growth stages. **a**. Principal component analysis of the RNA-Seq data. **b**. Four gene clusters with different expression patterns. Overlaying curves of all genes within the cluster were shown. **c**. Functional annotation of each gene cluster

Principal component analysis revealed that the samples of six stages with three biological replicates could be clearly assigned to four groups, referred to as S1, S2–1, S2–2, and S2–3 (Fig. 2a). The HY stage was found to group into cluster S1, with the other stages grouping together into cluster S2, indicating that the expression pattern in the asexual hyphae stage (HY) is different from the other stages. Of the five stages other than HY, the PR and MF stages grouped together and the YF and DF stages grouped together, while the ST stage remained separate, indicating that the DF and YF stages and the MF and PR stages have the more similar expression patterns.

The expression patterns of all the genes were further investigated through standardized Euclidean distance. The results demonstrated that there were four gene clusters with visible difference expression patterns (Fig. 2b). Cluster 1, with 112 transcripts, had a sudden increase in expression upon transition from the HY to the ST phase and then a gradual decrease in expression upon shifting from the PR to the MF phase. Cluster 2, with 1691 transcripts, had a gradual decrease in expression over the entire development process, illustrating a different role for these genes with respect to development. Based on the functional annotations of the gene clusters, cluster 1 mainly consisted of autophagy and nucleotide excision repair genes, while cluster 2 was composed of genes related to the biosynthesis of secondary metabolites, antibiotics and amino acids (Fig. 2c). Cluster 3 had the greatest number of genes (6129); their annotated functions were related mainly to RNA transport and endocytosis, and their expression showed a steady trend in all growth stages (Fig. 2b, c). By contrast, cluster 4 contained the least genes (7). Their expression was increased from the HY to the ST stage and from the PR to the YF stage and decreased from the ST to the PR stage and from the DF to the MF stage, while their expression remained steady from the YF to the DF stage (Fig. 2b).

### Identification of differentially expressed genes across various developmental stages

**Fig. 3:**
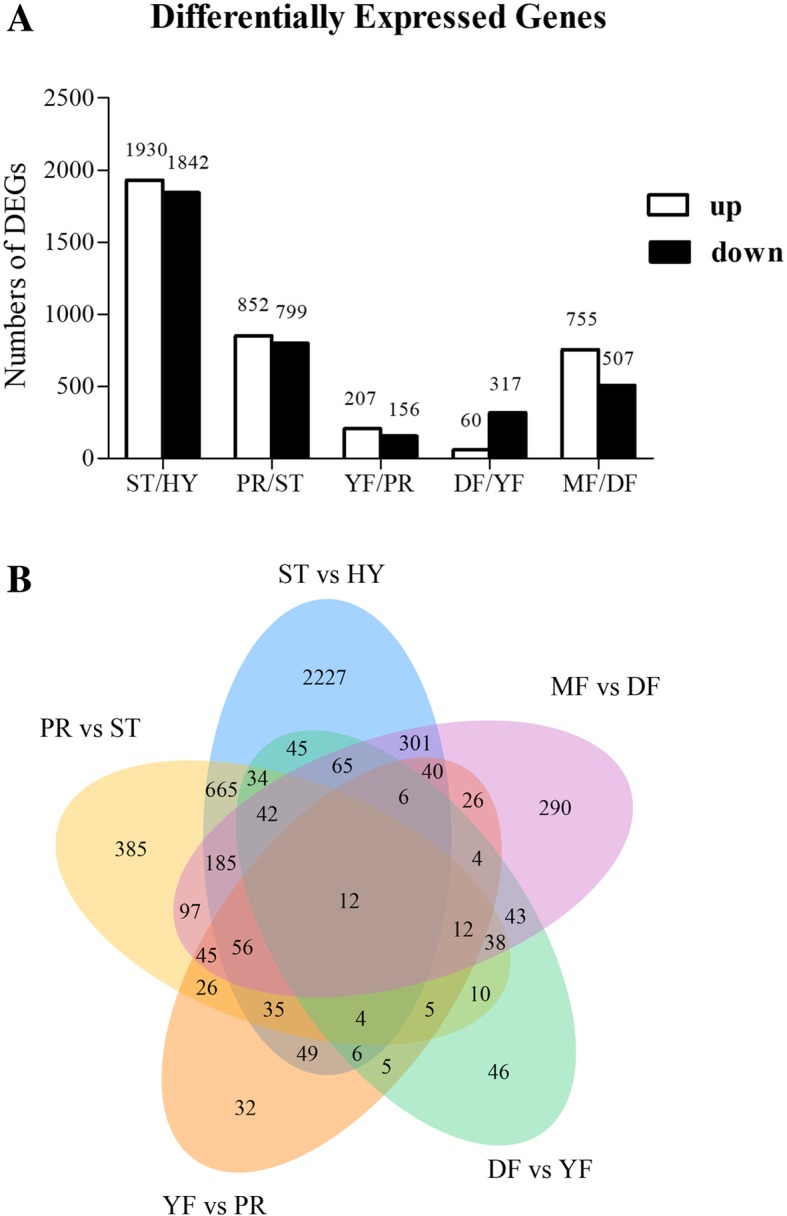
Analysis of DEGs between adjacent growth stages. **a**. DEG distribution between two samples analyzed. The number of genes differentially expressed is indicated on the top of the histograms. **b**. Venn diagrams comparing shared DEGs between the adjacent growth stages

Differentially expressed genes (DEGs) across consecutive developmental stages were identified based on FPKM values with a corrected *P*-value of 0.005 and a |log_2_ (Fold Change)| of 1 set as thresholds. As shown in Fig. 3a, the largest number of DEGs were identified between the ST and HY stages (3772), covering 47.5% of the *O. sinensis* genome, followed by PR vs. ST (1651) and MF vs. DF (1262) (Fig. 3a). Significantly fewer DEGs were identified in the comparisons between the YF and PR stages (363) and the DF and YF stages (377). These results revealed that the expression profiles of the primordium, young fruiting body and developed fruiting body stages are similar with only minor differences. The greatest number of unique DEGs were found in the comparison between the ST and HY stages (2227), while only 32 DEGs were unique to the YF vs. PR comparison (Fig. 3b). There were 12 shared DEGs among all five comparisons of adjacent growth stages (Additional file 1: Table S2) and these were enriched primarily with the response to oxidative stress and peroxidase activity.

### Functional classification of differentially expressed genes

**Fig. 4:**
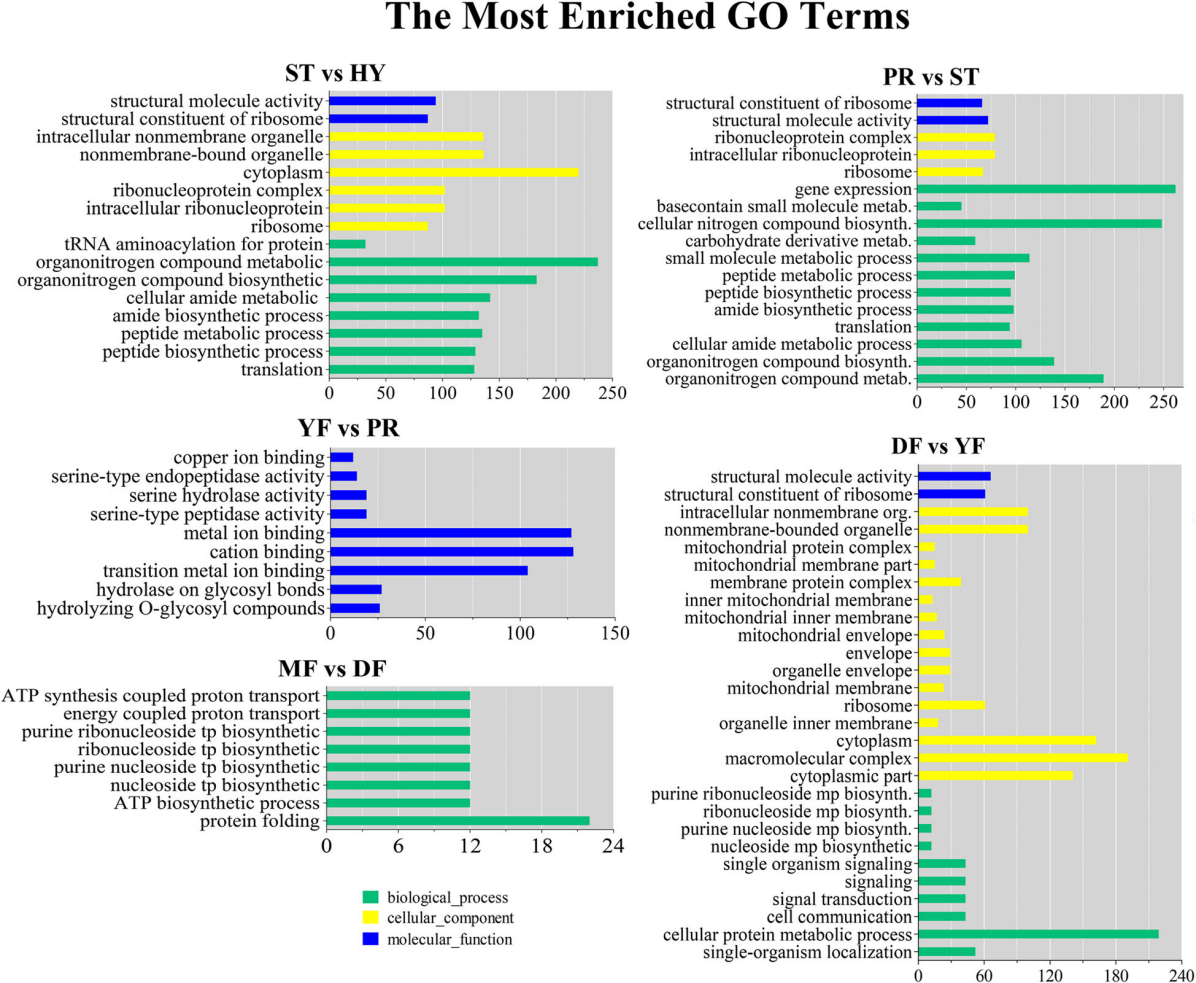
GO functional classification of differentially expressed genes. The green bars represent biological processes; yellow bars represent cellular components; blue bars represent molecular functions. Only the significant Go terms (*P* < 0.005) were shown

All DEGs were classified into three different categories: biological process (BP), cellular component (CC) and molecular function (MF). The top 10 significantly enriched terms were almost the same for the ST vs. HY and PR vs. ST comparisons (Fig. 4 and Additional file 1: Table S3). All of the significantly enriched terms for the YF vs. PR comparison were assigned into the molecular function category and included hydrolase activity, metal ion binding and serine-type peptidase activity. All of the significantly enriched terms for the MF vs. DF comparison were assigned into the BP category and included protein folding (GO: 0006457), ATP synthesis (GO: 0006745, 0015986) and nucleoside biosynthesis (GO: 0009142, 0009145, 0009201 and 0009206).

The DEGs were mapped to the Kyoto Encyclopedia of Genes and Genomes (KEGG) database and tested for enrichment to further research their functions. The DEGs from the ST vs. HY, PR vs. ST, YF vs. PR, DF vs. YF and MF vs. DF comparisons belonged to 12, 8, 4, 2 and 4 significantly enriched pathways, respectively (Additional file 1: Table S4). The ribosome pathway (cmt03010) was the most cohesive pathway, with a *P*-value of nearly 0 in the ST vs. HY, PR vs. ST and DF vs. YF comparisons. The ribosome pathway is composed of genes that encode the various proteins that make up the ribosomal subunits. The most significantly enriched pathways for the YF vs. PR and MF vs. DF comparisons were starch and sucrose metabolism (cmt00500) and inositol phosphate metabolism (cmt00562), respectively.

### Gene coexpression networks construction

**Fig. 5:**
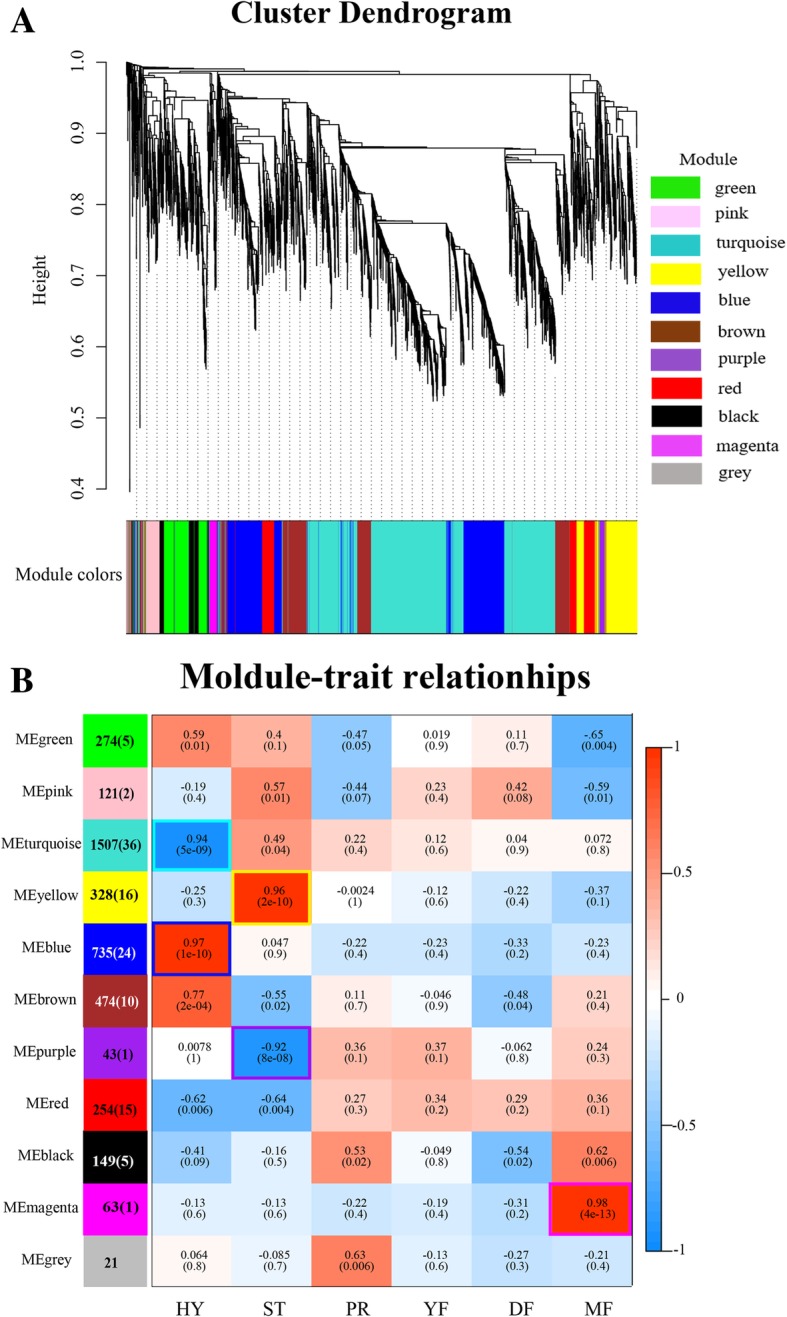
WGCNA of genes in different developmental stages in Chinese cordyceps. **a**. Hierarchical cluster tree showing coexpression modules identified by WGCNA. Each leaf on the tree represents one gene. The major tree branches constitute 11 modules, labeled with different colors. **b**. Relationships of consensus module eigengenes and different stages. Each row in the table corresponds to a consensus module, labeled with a color as in (**a**) and each column represents a developmental stage. The module name is shown on the left side of each cell. The number of genes in each module is indicated on the left and the number of TFs in each module is indicated by the number in parentheses. Numbers in the table report the correlations of the corresponding module eigengenes and stages, with the *P*-values printed below the correlations in parentheses. Each column corresponds to a specific stage. Scale bar on the right indicates the range of possible correlations from positive (red color, 1) to negative (blue color, − 1). The boxes with a colored outline corresponding to the module indicated the significant correlation between the module eigengene and the stage

To obtain a comprehensive understanding of the genes expressed in the successive developmental stages, a WGCNA was performed [15]. Clusters of highly correlated genes with high correlation coefficients were defined as modules. This analysis identified 11 different modules (marked with different colors, Fig. 5a), in which the major tree branches define the modules. The 11 modules correlated with different development stages due to stage-specific expression profiles.

The blue module identified 735 genes specific to the HY stage (Fig. 5b), including 24 transcription factors (TFs). Among them, 11 significant pathways were enriched (Additional file 1: Table S5). All of the 11 significant pathways were metabolism-related, including primary metabolism pathways (carbon metabolism, starch and sucrose metabolism, fructose and mannose metabolism, cysteine and methionine metabolism, beta-alanine metabolism, etc.) and secondary metabolism pathways (biosynthesis of secondary metabolites, biosynthesis of antibiotics, etc.). The most enriched pathway was steroid biosynthesis (Additional file 1: Table S5). The 24 TFs encode 12 Zn2Cys6 TFs, 3 homeodomain-like TFs, 3 winged helix repressor DNA-binding TFs, and 3 ZIP TFs, among others (Additional file 1: Table S6). The turquoise module, representing 1507 genes, was highly negatively associated with the HY stage and was also enriched mainly in metabolism-related pathways (Fig. 5b and Additional file 1: Table S5). There were 36 TFs, including 11 Zn2Cys6 TFs and 4 C2H2 zinc finger TFs, among others (Additional file 1: Table S6).

The yellow module, with 328 identified genes, was highly associated with the ST stage (Fig. 5b). The significantly enriched pathways included xenobiotics biodegradation and metabolism (atrazine degradation), amino acid metabolism (alanine, aspartate and glutamate metabolism, and arginine biosynthesis), lipid metabolism (ether lipid metabolism) and carbohydrate metabolism (butanoate metabolism) (Additional file 1: Table S5). There were 16 genes encoding TFs in the yellow module, including 8 Zn2Cys6 TFs and 3 winged helix repressor DNA-binding TFs (Additional file 1: Table S6). The purple module, containing 43 genes, was negatively correlated with the ST stage (Fig. 5b). The only significantly enriched KEGG pathway was RNA transport with only one TF-encoding gene, the Grainyhead/CP2 TF (Additional file 1: Table S5, S6).

The magenta module (63 genes) was highly associated with the MF stage (Fig. 5b) and was enriched for the pentose phosphate pathway, tryptophan metabolism, biosynthesis of antibiotics and carbon metabolism. Only one TF, HMG (Additional file 1: Table S5, S6), was included in the magenta module.

The module significance (MS), which is determined as the mean gene significance (GS) across all the module genes, were used to evaluate the association between modules and phenotypes. For each module, we plotted scatter plots of GS vs. MM (module membership) (Additional file 2: Figure S3). It can be seen that the GSs in the blue, yellow and magenta modules is highly positively correlated with MM (correlation coefficient = 1, *p* < 1^e-200^), illustrating that the modules were significantly associated with the HY, ST, and MF stages, respectively. The GSs in the turquoise and purple modules were negatively correlated with the HY and ST stages (correlation coefficient = − 1, *p* < 1^e-200^), respectively.

WGCNA can also be employed to construct gene networks in which each node represents a gene and the connecting lines (edges) between genes represent coexpression correlations [15]. The coexpression networks of the top ranked genes for five selected modules, including the blue, yellow, turquoise, purple and magenta modules, were constructed as shown in Additional file 2: Figure S4. Those genes which showed the most interconnections in the network were identified as hub genes, as indicated by their high KME (eigengene connectivity) value. The top ten genes with the highest KME values in each of the specific modules are shown in Table 1.

**Table 1:** Candidate hub genes in HY, ST and MF stages

Gene name	Description	kME value
HY-specific blue module		
OSIN7260	Hypothetical protein	0.9860
OSIN3519	Hypothetical protein	0.9845
OSIN3540	Glycosyl transferase	0.9844
OSIN6821	Bicarbonate transporter	0.9842
OSIN3078	Hypothetical protein	0.9826
OSIN6482	Hypothetical protein	0.9821
OSIN6169	Hypothetical protein	0.9818
OSIN3902	Oxidoreductase	0.9810
OSIN6907	Hypothetical protein	0.9806
OSIN6949	Hypothetical protein	0.9806
HY-specific turquoise module		
OSIN6101	Hypothetical protein	0.9946
OSIN1039	Hypothetical protein	0.9933
OSIN5827	E1-like protein-activating enzyme Gsa7p/Apg7p	0.9929
OSIN3683	Hypothetical protein	0.9921
OSIN3387	Meiotically up-regulated protein	0.9914
OSIN4246	WD domain, G-beta repeat containing protein	0.9914
OSIN1746	Hypothetical protein	0.9913
OSIN0373	Zn2Cys6 transcription factor, fungi	0.9910
OSIN0998	START-like domain protein	0.9905
OSIN6059	Stress-responsive protein Ish1	0.9905
ST-specific yellow module		
OSIN1750	Low temperature requirement A	0.9878
OSIN1054	TBC1 domain family member 5	0.9843
OSIN0446	N-terminal fungal transcription regulatory domain-containing protein	0.9823
OSIN1014	Peroxin-3 family protein	0.9749
OSIN3726	Cytochrome P450	0.9707
OSIN1666	Dynactin subunit	0.9704
OSIN6003	Hypothetical protein	0.9695
OSIN3725	Hypothetical protein	0.9650
OSIN0445	Basic-Leucine zipper (bZIP) transcription factor	0.9638
OSIN1672	Terpenoid synthase	0.9608
ST-specific purple module		
OSIN3113	N-acetyltransferase complex ard1 subunit	0.9877
OSIN4003	Clathrin adaptor complex	0.9681
OSIN6067	Hypothetical protein	0.9680
OSIN7385	Hypothetical protein	0.9651
OSIN2377	NAD(P)-binding domain protein	0.9570
OSIN4113	Six-bladed beta-propeller, TolB-like protein	0.9528
OSIN3236	Peroxisomal biogenesis factor 2	0.9506
OSIN1470	Benzoyl formate decarboxylase	0.9506
OSIN6963	Putative methyltransferase	0.9484
OSIN4021	UTP-glucose-1-phosphate uridylyltransferase	0.9445
MF-specific magenta module		
OSIN3310	Non-Catalytic module family expansin	0.9791
OSIN6011	Hypothetical protein, only exist in *Ophiocordyceps sinensis*	0.9745
OSIN3409	Peptidase A1	0.9622
OSIN0493	Hypothetical protein	0.9544
OSIN3601	Hypothetical protein	0.9543
OSIN0492	Hypothetical protein, only exist in *Ophiocordyceps sinensis*	0.9469
OSIN1023	Hypothetical protein	0.9467
OSIN1456	Hypothetical protein	0.9409
OSIN2949	Methyltransferase type 11	0.9331
OSIN4742	Major facilitator superfamily domain, general substrate transporter	0.9283

**Fig. 6:**
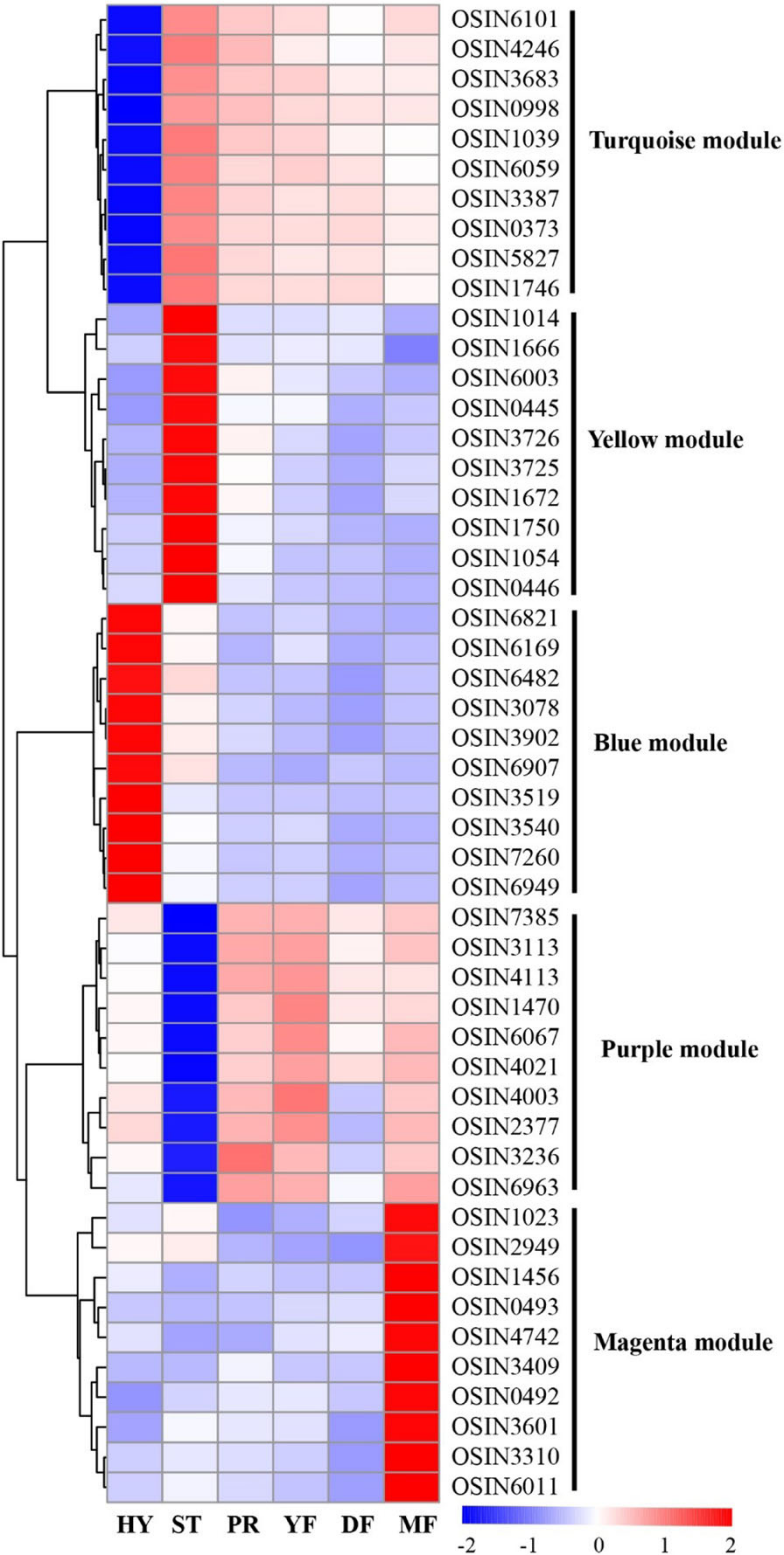
Heatmap clustering of top ten candidate hub genes in the HY, ST and MF stages. The levels of expression are represented by log_2_(FPKM + 1) values after centralization correction. Genes with similar patterns of expression are clustered together

The HY stage-specific blue module genes were overrepresented in the HY stage (Fig. 6). Among the 10 candidate hub genes of the HY stage-specific blue module, 7 were hypothetical proteins and the other three were a glycosyl transferase, a bicarbonate transporter and an oxidoreductase (Table 1). The HY stage-specific turquoise module genes were lowly expressed in the HY stage (Fig. 6). Candidate hub genes identified for the HY stage-specific turquoise module (negatively correlated with HY stage) included the E1-like protein-activating enzyme Gsa7p/Apg7p, a meiotically upregulated protein, a WD domain protein, a G-beta repeat containing protein, a START-like domain protein, the stress-responsive protein Ish1, a Zn2Cys6 TF and 4 hypothetical proteins.

The ST stage-specific yellow module genes were overrepresented in the ST stage (Fig. 6). The candidate hub genes included low temperature requirement A, TBC1 domain family member 5, N-terminal fungal transcription regulatory domain-containing protein, peroxin-3 family protein, Cytochrome P450, dynactin subunit, bZIP TF, terpenoid synthase and 2 hypothetical proteins. The ST stage-specific purple module genes were lowly expressed in the ST stage (Fig. 6). Genes identified as candidate hub genes included the N-acetyltransferase complex ard1 subunit, clathrin adaptor complex, NAD(P)-binding domain protein, TolB-like protein, peroxisomal biogenesis factor 2, benzoyl formate decarboxylase, putative methyltransferase, UTP-glucose-1-phosphate uridylyltransferase and 2 hypothetical proteins.

The MF stage-specific magenta module genes were overrepresented in the MF stage (Fig. 6). Among the 10 candidate hub genes, 6 were hypothetical proteins and the other four included a noncatalytic module family expansin, peptidase A1, methyltransferase type 11, a major facilitator superfamily domain protein, a general substrate transporter and two hypothetical proteins. In particular, the two hypothetical proteins (OSIN6011 and OSIN0492) only exist in *O. sinensis*, and there are no homologues in the other fungi.

### Differential expression of mating-type genes

Sexual development in ascomycetes is controlled by the (MAT) locus, which regulates the production of the fruiting body [20, 21]. Previous analysis of the mating-type genes in *O. sinensis* revealed the presence of three genes (MAT1–1-1, MAT1–1-2 and MAT1–1-3) in the MAT1–1 idiomorph and a single gene (MAT1–2-1) in MAT1–2 idiomorph [14, 22]. It was found that these mating type loci genes were differentially expressed, with very low expression (read count of 0–440 and FPKM of 0–18.4) among the different stages. None of the three genes in the MAT1–1 idiomorph were expressed in the HY stage (read counts = 0). MAT1–1-1 (OSIN7648) was expressed in the other five stages and had a relatively high expression level in the PR and MF stages. MAT1–1-2 (OSIN7647) was only expressed in the PR and MF stages, with very low expression (read counts = 1.18, 10.67 and FPKM 0.04, 0.40, respectively). MAT1–1-3 (OSIN7646) was expressed in all stages other than the HY and ST stages. MAT1–2-1 (OSIN7649) was expressed in each stage, but with very low read counts of 1–18 (FPKM 0.16–0.93). The three genes in the MAT1–1 idiomorph were up-regulated by 1.8, 6.1 and 3.7-fold, respectively when the MF stage was compared to the DF stage. However, there was no differential expression for the mating 1–2-1 (Additional file 1: Table S7). The expression of these mating genes was verified by semiquantitative PCR (Additional file 2: Figure S5).

**Fig. 7:**
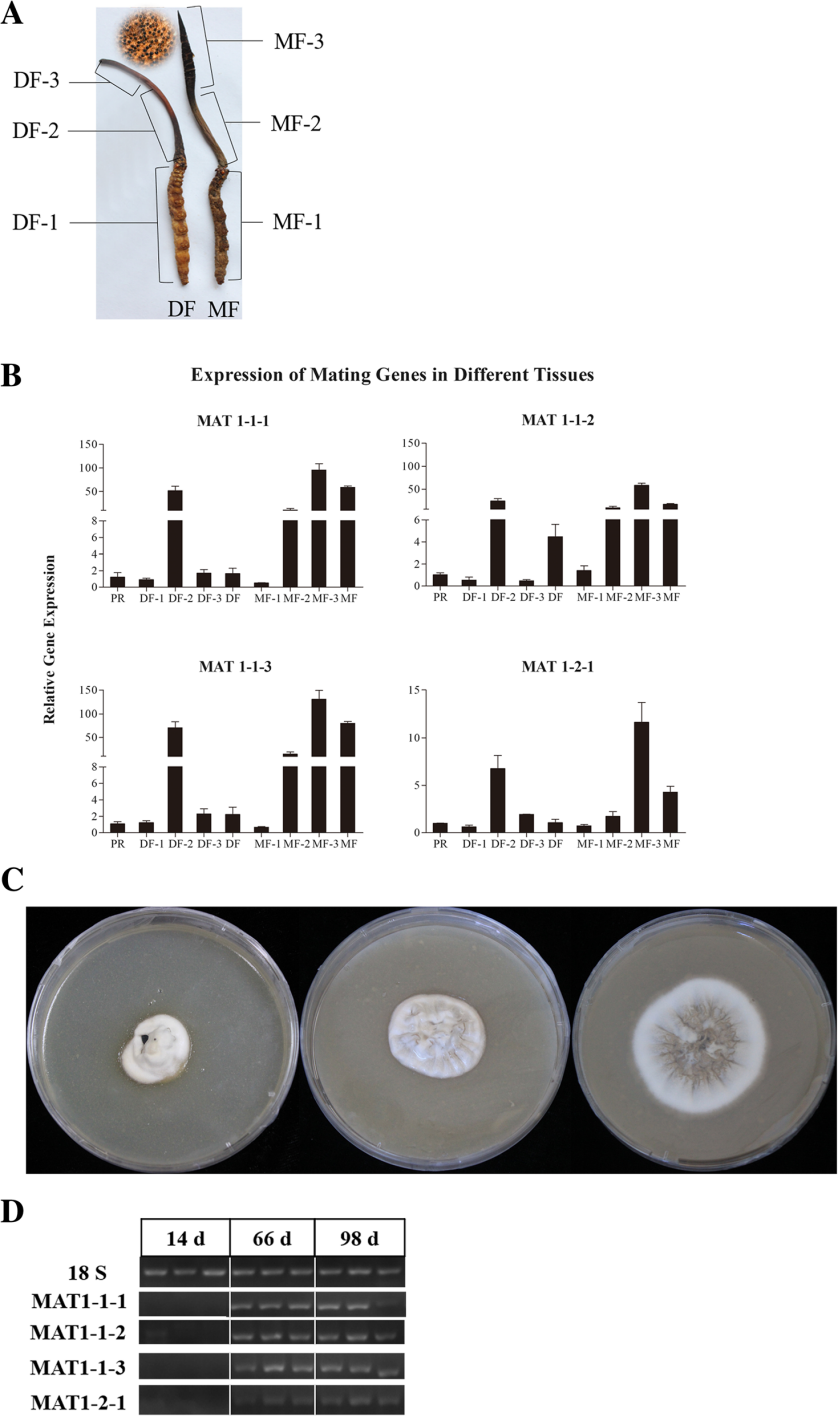
Expression of mating genes in different tissues of Chinese cordyceps and mycelia cultured on solid media for different amounts of time. **a**. Different tissues of Chinese cordyceps. MF-1 and DF-1, the sclerotium of MF and DF, respectively; MF-2, the stipe of the MF stroma; MF-3, terminal fertile part with superficial perithecia. The inner illustration is the perithecia on the surface of MF-3. DF-2 and DF-3 are the corresponding parts to MF-2 and MF-3, respectively. **b**. Expression of mating genes in different tissues of Chinese cordyceps by RT-qPCR. **c**. Colony of Chinese cordyceps cultured on solid media for different amounts of time. **d**. Semiquantitative RT-PCR analysis of mating genes in the mycelia cultured on solid media for different amounts of time

Chinese cordyceps is a parasitic complex that is made up of the remains of the caterpillar and fungal stroma. There are two distinct parts of the mature stroma, the sterility stipe (MF-2) and the terminal fertile part with a superficial perithecia (MF-3, Fig. 7a). The expression levels of the mating genes were tested in the different tissues during the DF and MF stages and compared with that of the PR stage, because all four mating genes are expressed in the PR stage. The expression of all four mating genes were found to be the lowest in the sclerotium of the DF (DF-1) and the MF (MF-1). All four mating genes were expressed the highest in the MF stage in the fertile part of the MF stroma (MF-3), which was much higher than in the sterility stipe (MF-2) (Fig. 7b, Additional file 2: Figure S7). However, the opposite results were obtained in the DF stage (Fig. 7b, Additional file 2: Figure S6). This result has been confirmed by repeated experiments.

A recent study reported that the mating type genes were barely detectable both before and after infection (both RPKM values = 0) and suggested that mating type genes did not participate in anamorphic hyphae amplification or yeast-like hyphal body multiplication [23]. However, the MAT1–1-1 gene expressed in the liquid medium of cultured anamorph mycelia [10]. We detected the expression of the mating type genes in mycelia growing on solid culture medium for different days (14 d, 66 d and 98 d) (Fig. 7c). No expression for any of the 4 mating type genes was found in 14 d, but the expression of all four genes were detected in the other timepoints (Fig. 7d).

### Differential expression of sexual development related genes

Fungal sexual development related genes that have been functionally verified in the model ascomycetes *Aspergillus nidulans* and *Neurospora crassa* [24] were used for blastp search against the genomes of *O. sinensis* to retrieve the respective homologs. The differential expressions of these 86 genes were analyzed (Additional file 1: Table S7).

**Fig. 8:**
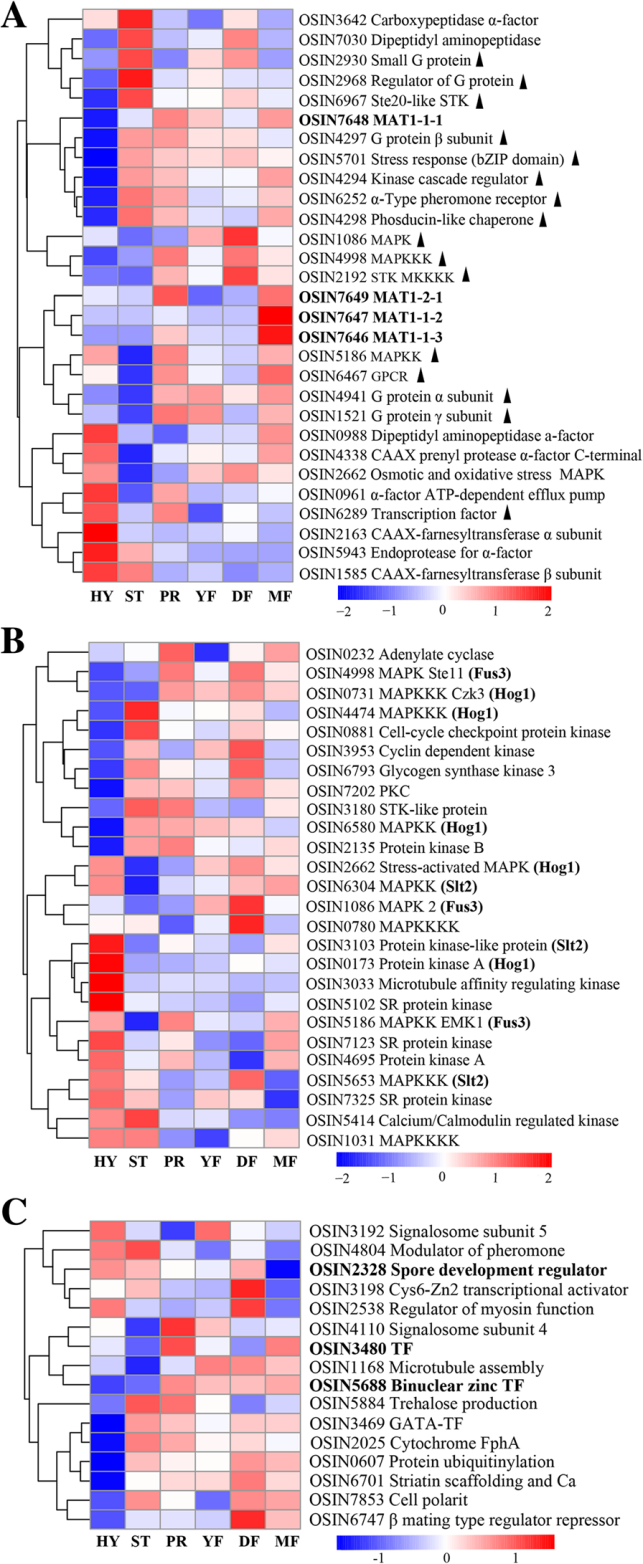
Expression of mating- and fruiting body development-related genes in different stages. **a**. Heatmap of mating process and mating signaling-related genes. The triangle indicated the genes related with mating signaling. **b**. Heatmap of protein kinase genes associated with the mitogen-activated and cAMP-dependent protein kinase pathways. **c**. Heatmap of fruiting body development related genes

Most of the genes related to mating signaling were generally expressed lower in the HY stage than in the other stages (Fig. 8a). Among them, half of them (OSIN 2930, 2968, 6967, 4297, 5701, 4294, 6252 and 4298) showed as the highest expression in the ST stage. None of the mating signaling genes were differentially expressed when the MF stage was compared with the DF stage or when the DF stage was compared with the YF stage.

Pheromone receptors control the fruiting body formation and sexual cycle in fungi, but not vegetative growth [25]. A pheromone receptor (OSIN6252) was expressed lowly in the HY stage and was upregulated significantly in the ST stage, after which its expression was slightly decreased until the MF stage (Additional file 1: Table S7). Compared to undifferentiated HY stage, OSIN6252 was significantly upregulated in all of the other stages. However, Pth11-like G-protein coupled receptor (GPCR; OSIN2887 and OSIN5308), orthologues of CCM_03015, which is a pheromone receptor significantly upregulated during the initiation of fruiting body formation in *C. militaris* [26], had almost steady expression during the entire developmental period.

The adenylate cyclase gene OSIN0232 was constitutively transcribed at a moderate level (FPKM 16–32) during the growth of *O. sinensis* (Fig. 8b, Additional file 1: Table S8). Protein kinase A (PKA; OSIN0173) was expressed highest in the HY stage, indicating its relation to vegetative growth. Another PKA gene (OSIN4695) had constitutive expression during the whole growth period (Fig. 8b, Additional file 1: Table S8). Other protein kinases such as OSIN 3103, 7325 and 7123 showed the highest expression in the HY stage (Fig. 8b, Additional file 1: Table S8). It seemed that the cAMP-dependent PKA pathway plays a minimal role in fruiting body initiation and development in Chinese cordyceps.

Mitogen-activated protein kinase (MAPK) genes are required for fruiting in some fungi [25] and we analyzed the gene expression of homologous of sexual development-related genes functionally verified in *Aspergillus nidulans* and *Neurospora crassa*. A total of nine MAPKKK/MAPKK/MAPK protein-encoding genes were reported in the *Neurospora* genome sequence [27]. The similarity scores demonstrate that these proteins existed in *O. sinensis* genome (Additional file 1: Table S8) and maybe also form three pathways corresponding to those for Fus3 (pheromone response), Hog1 (Osmosensing and stress), and Slt2 (cell integrity pathways) in *Neurospora*. OSIN4998 (MAP kinase kinase kinase Ste11)/OSIN1086 (MAP kinase 2)/OSIN5186 (MAPKK, Ste. 7) were the orthologous protein of the of *N. crassa* Fus3 cascade required for the pheromone response. Among them, the expression of OSIN4998 and OSIN1086 had the constitutive expression during the growth period. However, the expression of OSIN5186 was upregulated significantly in the PR, YF, DF and MF stages when compared with ST stage (Fig. 8b, Additional file 1: Table S8). OSIN6580 and OSIN4474, orthologous protein of *N. crassa* Hog1 cascade involved in adaptation to high osmotic pressure [28], showed the highest expression in the ST stage. The three genes of orthologous proteins of *N. crassa* Slt2 cascade (OSIN5653/OSIN6304/OSIN3103), is known to have a role in the cell wall integrity signaling pathway [29], showed the highest expression in the vegetative growth HY stage.

The calcium/calmodulin-regulated kinase which was sharply upregulated in the fruiting body development stage in *C. militaris* [26], had relatively high expression in the HY and ST stages and then decreased significantly in the other stages (CaMK, OSIN5414). Protein kinase C (OSIN7202), whose orthologous gene is almost not transcribed during the growth of *C. militaris* [26], was expressed during all growth stages and was upregulated during the fungal fruiting stages (ST, PR, YF, DF, MF) compared to the undifferentiated HY stage.

The genes known to be necessary for fruiting body development (in the model fungi *A. nidulans* and *N. crassa*) (Additional file 1: Table S7) were expressed at each growth stage. When comparing the PR and ST stages, two transcriptional activators (OSIN5688 and OSIN3480) were differentially expressed (*P*-value < 0.005). OSIN5688 was expressed at a much higher level in the fruiting body developmental stages, including PR, YF, DF and MF, compared to the HY and ST stages, reaching its highest expression in the PR stage (Fig. 8c). When comparing the MF and DF stages, the velvet activator gene, VeA (OSIN2328), was significantly downregulated and the transcription factor (OSIN3480) was significantly upregulated (Fig. 8c).

## Discussion

As one of the most valued traditional Chinese medicinal fungi, Chinese cordyceps was cultivated artificially in large-scale recently over several decades of exploration. However, the induction of primordium, the sexual reproduction and the molecular basis of its lifestyle still remain cryptic. Developmental transcriptomics can benefit our understanding of the biology of this fungus. In the present study, the transcriptomes of different developmental stages of Chinese cordyceps were compared using the same batch of samples and covering the entire developmental process. From this analysis, modules of coexpressed genes and candidate hub genes for each developmental stage were identified using WGCNA. Of particular focus were the spatiotemporal expression patterns of sexuality-related genes. The gene expression profiles during the primordium formation and fruiting body development stages were found to be more similar than the undifferentiated HY stage. Spatiotemporal specificity indicated that mating type genes express during both the fruiting body development stage and in the undifferentiation HY stage, and the 4 mating type genes have divergent roles. Half of genes related to mating signaling showed as the highest expression in the ST stage indicating fruiting in this fungus is initiated in the ST stage.

The samples used are critical for developmental transcriptome analysis. A transcriptome study was previously performed; however, the samples used were fresh fruiting bodies of wild *O. sinensis* sourced from herb market, which can’t cover the developmental stages [9, 10]. Another transcriptome analysis revealed the genes putatively related with mating and fruiting body development, and identified the putative enzymes involved with the biosynthesis of cordycepin, one of the medicinal compounds, and developed a model for the synthesis pathway [9]. However, homologous genes related with cordycepin biosynthesis which has been confirmed in *Cordyceps militaris* did not exist in the *O. sinensis* genome, it was believed that *O. sinensis* could not produce cordycepin [7, 30]. The differently expressed genes of *O. sinensis* growing for different periods were also determined and analyzed [31]; however, there was only an anamorph sample. The success of the artificial cultivation of Chinese cordyceps provides the possibility for research on developmental biology. The six samples included in our study (i.e., hyphae (HY), sclerotium (ST), primordium (PR), young fruiting body (YF), developed fruiting body (DF) and mature fruiting body (MF) covered the whole developmental process and all the samples were from the same batch of host, fungi and infection, which guarantee the reliability of the results.

As we expected, principal component analysis revealed that the gene expression profiles at the stages of primordium formation and fruiting body development were more closely related to each other than the vegetative HY stage (Fig. 2a), which is consistent with previous reports [10]. The fruiting body growth stages, YF and PR, and DF and YF, had the fewest differentially expressed genes (Fig. 3a), which also confirmed the observed similar gene expression. Mycelium form primordia which develop into young fruiting bodies, and then mature to release ascospores. The PR and MF stages grouped together (Fig. 2a), suggesting that the initiation of fruiting body development and the maturation of the ascospore have similar patterns of expression in this fungus.

More genes were differentially expressed from the vegetative HY stage to the ST stage, followed by the comparisons between the ST and PR stages and the DF and MF stages (Fig. 3a). The DEGs that were unique to comparisons between adjacent stages had the same trend of expression (Fig. 3b). In general, the particularly upregulated genes in undifferentiated HY stage were mainly related to carbohydrate metabolism and rapid growth.

Stage-specific modules and hub genes were identified using WGNCA. The blue and turquoise modules (positive and negative) included 735 and 1507 genes, respectively, that were highly associated with the HY stage (Fig. 5b). Almost all of the HY stage-specific enriched gene pathways were metabolism related, clearly indicating their importance in active cell division and the utilization of assimilates in the mycelia. Steroid biosynthesis was the most enriched pathway, consistent with the fact that the biomass increases in the HY stage (Additional file 1: Table S5). Glycosyl transferases (OSIN3540), being important for the synthesis of complex and biologically important carbohydrates [32], was one of the candidate hub genes for the HY stage.

The yellow and purple modules were highly associated (positively and negatively) with the ST stage (Fig. 5b) and amino acid, lipid and carbohydrate metabolism were enriched, indicating that cell division is still active in the ST stage. Importantly, one cytochrome P450 (OSIN3726), a heme-containing monooxygenase, was identified as one of the hub genes. Studies in the plant pathogen *Fusarium graminearum* indicated that cytochrome P450 is not only involved in secondary metabolism but is also required for fungal development and virulence [33]. The peroxin-3 family protein (OSIN1014), involved in peroxisome biosynthesis and integrity [34], was also the candidate hub gene. In fact, peroxidase activity, which acts as a ROS-scavenging enzyme, was demonstrated to have significant and positive correlations with fungal pathogenicity in the plant pathogenic fungus *Magnaporthe oryzae* [35]. The relationship between peroxidase activity and the infection of this fungus is the focus of another project in our laboratory.

Additionally, the fact that the pentose phosphate pathway was enriched in the MF stage (magenta module) is noteworthy. The pentose phosphate pathway is a metabolic pathway that parallels glycolysis, producing energy for normal growth and differentiation and reducing compounds, such as NADPH, for biosynthesis as well as for ribose 5-phosphate. It has also been reported that the pentose phosphate pathway is related to the teleomorph stage in some fungi. Radio respirometry and enzymological analysis of carbohydrate metabolism in different tissues of *Lentinula edodes* revealed that metabolic activity through the pentose phosphate pathway was very high in the basidium, but low in mycelium; additionally, the activity of PPP enzymes (glucose 6-phosphate dehydrogenase and 6-phosphogluconate dehydrogenase) were three times higher in the basidium compared to the mycelium [36]. Comparative proteomic analysis during asexual and sexual spore development of *Aspergillus cristatus* revealed that the energy compounds produced from carbon metabolic pathways were mainly in the form of ATP and NADH in the agamotype, whereas they were NADPH and FAD in the teleomorph [37].

Almost half of the candidate hub genes are functionally uncharacterized, suggesting that genes coding for unknown proteins may be more related to developmental processes. These unknown genes would be the targets for future researches.

There was little or no expression in any of the growth stages for all four mating genes. Zhang et al. [11] also reported the low expression of mating genes in wild organisms collected from the Qinghai-Tibetan plateau with FPKM values of 0–2. Our study also found that these mating genes were differentially expressed between the undifferentiated HY stage and the teleomorph stages.

Previous studies have indicated that the functions of the MAT genes go beyond sexual development [38, 39]. It was reported that none of the mating type genes were expressed in solid medium-cultured anamorph mycelia and hyphal bodies (read counts = 0) before and after *O. sinensis* infected the host larvae [23]. Our transcriptome results confirmed that the three MAT1–1 genes were not expressed in solid medium-cultured mycelia, and it seemed that they did not participate in anamorphic hyphae amplification of *O. sinensis*. However, transcriptional profiling of the mycelia cultured for different days on solid media confirmed that the expression of the 4 mating genes could be detected when cultured for relatively long time (Fig. 7d). The mating type genes (MAT1–1-1. MAT1–1-2 and MAT1–2-1) of *C. militaris* were reported to be expressed in Sabouraud dextrose broth [40].

Xiang et al. [9] reported that the transcripts for all the mating genes were expressed in fresh fruiting bodies of wild *O. sinensis* sourced from an herb market. Our results found that these four genes were only expressed simultaneously in the PR (primordium differentiation) and MF (sexual maturation) stages. The three MAT1–1 genes were not coordinately expressed, suggesting that they may have divergent roles, which has been demonstrated in the related fungus *C. militaris* [40].

Analysis of the expression in different tissues of Chinese cordyceps indicated that all four mating genes were expressed at much lower levels in the sclerotium of the DF (DF-1) and MF (MF-1) stages compared to the stroma parts. This suggested that the sclerotium may only supply the nutrients for sexual reproduction. In the MF stage, all four mating genes were expressed the highest in the fertile part of the MF stroma (MF-3) and this was much higher than in the sterility stipe (MF-2) (Fig. 7b, Additional file 2: Figure S6), indicating that the 4 genes participate in sexual maturation. In the DF stage before the sexual maturation, the opposite results were obtained, with the expression of the mating type genes much higher in the sterility stipe (DF-2) than in the DF-3 (Fig. 7b, Additional file 2 Figure S6), indicating that the expression of mating type genes may pass some signals involved in sexual maturation to the upper during the fruiting body development. Divergent spatial distribution of the mating type genes in morels has also been reported [41]; however, this is the first report to our knowledge of the spatial specificity of mating type gene expression.

In response to extracellular stimuli, two cytoplasmic signaling branches defined by cAMP-dependent PKA and MAPKs regulate gene expression that ultimately leads to ascocarp formation in Ascomycetes [42]. Components of the cAMP pathway were shown to influence sexual development in *Trichoderma reesei* [43] and homothallic *Sordaria macrospora* [44]. In the plant pathogen *Magnaporthe grisea*, it was shown that the sexual cycle is dependent on cAMP [25], but fruiting by *C. militaris* in the absence of a partner is more dependent on the MAPK pathway than the cAMP-dependent PKA pathway [26]. Based on the constitutive expression of the only adenylate cyclase (OSIN0232) and PKA (OSIN4695), it appears as though the fruiting body development of Chinese cordyceps is not dependent on the cAMP-dependent PKA pathway. Half of genes related to mating signaling showed as the highest expression in the ST stage, indicating fruiting in this fungus is initiated in the ST stage.

MAPK pathways consist of three serine/threonine protein kinases (MAPKKK, MAPKK, and MAPK) that act sequentially, culminating in phosphorylation of target proteins that regulate transcription, the cell cycle, or other cellular processes [45]. Three major classes of MAPKs being homologous to the yeast Hog1, Fus3 and Slt2 have been identified in the filamentous fungi. They have been reported to related with the high osmotic pressure adaption [28], pheromones response and induction of differentiation processes which can trigger sexual reproduction [46], and playing a role in the signaling pathway of cell wall integrity in fungi [29]. 203-20A total of ten MAPKKK/MAPKK/MAPK protein-encoding genes were identified in the genome of *O. sinensis* (Additional file 1: Table S8). Mutations of both mak-2 (NCU02393) and MAPKK kinase (NCU06182), genes of Fus3 cascade in *N. crassa* resulted in inappropriate conidiation, female sterility, and loss of hyphal fusion [27]. OSIN4998 (MAP kinase kinase kinase Ste11), OSIN5186 (MAP kinase kinase) and OSIN1086 (MAP kinase 2) are the orthologous proteins of *N. crassa* Fus3 cascade required for the pheromone response. The upregulate expression of OSIN5186 (the orthologous protein of the of *N. crassa* Fus3- MAPKK) significantly in the PR, YF, DF and MF stages indicated its relation with the sexual development. The activated MAPK can activate TFs and regulate mating processes and fruiting body development (Additional file 2: Figure S8). The high expression of genes of orthologous protein *N. crassa* Hog1 cascade (OSIN6580 and OSIN4474) in the ST stage indicated that Hog1-MAPK may response to the biological stress in *O. sinensis.*

At the same time, the beta-subunit G protein (OSIN4297), a pheromone receptor (OSIN6252) had the highest expression in the ST stage and then remained at a relatively highly expression level (Additional file 1: Table S7), suggesting that fruiting in this fungus is initiated in the ST stage.

## Conclusions

Our results provide the insight into the key pathways and hub genes, and sexuality-related gene profile in the development of Chinese cordyceps from the transcriptomic perspective. It was verified that transcriptional profiles at the stages of fruiting body development more closely resembled each other than that of undifferentiated HY stage. Mating type genes may function at both the fruiting body development stage and the undifferentiation HY stage, and there is spatial specificity for the expression of 4 mating type genes. This is the first detailed developmental transcriptomic study of this important fungus and it will be helpful in understanding fruiting body formation and sexual reproduction and provide a foundation for the improvement of the artificial cultivation of this fungus.

## Methods

### Collection of fungal materials at different developmental stages

Six developmental stages of Chinese cordyceps (Fig. 1a) were harvested from the artificial cultivation workshop at Sunshine Lake Pharma Co. Ltd. The hyphae were cultured on potato dextrose agar supplemented with 5% wheat bran and 0.5% peptone and were designated as hyphae (HY). The mummified larvae coated with mycelia before stroma development were designated as the sclerotium (ST). The samples of stroma with lengths < 1 cm, 1–3 cm and 5 cm were designated as the primordium (PR), young fruitbody (YF) and developed fruiting body (DF), respectively. The fruiting body with mature perithecia and ascospores was the sixth sample and was designated as the mature fruiting body (MF) (Fig. 1b).

### RNA isolation, library construction and sequencing

The total RNA was extracted using the E.Z.N.A. TM Plant RNA Kit (Omega, Stamford, CT, USA). RNA quality and concentration were evaluated by the RNA Nano 6000 Assay Kit of the Bioanalyzer 2100 system (Agilent Technologies, CA, USA) and the Qubit® RNA Assay Kit on the Qubit® 2.0 Fluorometer (Life Technologies, CA, USA). Sequencing libraries were generated using an NEBNext® Ultra TM RNA Library Prep Kit for Illumina® (NEB, Ipswich, MA, USA) following the manufacturer’s recommendations. The library quality was assessed on an Agilent Bioanalyzer 2100 system. The cDNA libraries were sequenced on an Illumina HiSeq 2000 platform at Novogene Bioinformatics Technology Co., Ltd. (Beijing, China). Clean data or clean reads were obtained by removing reads containing the adapter or ploy-N sequences, as well as removing low quality reads from the raw data. All of the following of the analyses were based on this clean data.

### Alignment with the reference genome and differentially expressed gene analysis

The genome sequences (Accession: PRJNA382001) and annotation files of *O. sinensis* were downloaded from the following platforms at: http://www.plantkingdomgdb.com/Ophiocordyceps_sinensis/ [8]. This assembly contains 156 scaffolds (> 2 Kb; N50 = ~ 3.0 Mb), has a length of ~ 116.4 Mb, and predicted 7939 protein-coding genes [8]. Clean reads from each sample were mapped to the reference genome using HISAT2 (v2.0.5) [47]. The transcript reconstruction was carried out with the software Cufflinks together with TopHat2 [48].

Gene expression levels were normalized using the FPKM (Fragments per kilobase of transcript million mapped reads) method. The DESeq2 R package (1.16.1) [49] was used to identify differentially expressed genes (DEGs) across samples. The *P*-values were adjusted using the method of Benjamini and Hochberg [50]. Genes with a fold change ≥1 and a false discovery rate (FDR) < 0.005 in a comparison were identified as significantly DEGs. Enrichment analysis for Gene Ontology (GO) functions and Kyoto Encyclopedia of Genes and Genomes (KEGG) pathways were then performed. Raw Illumina sequencing data of *O. sinensis* were submitted to NCBI as BioProject GSE123085.

### Functional annotation of DEGs

GO enrichment analysis of differentially expressed genes was implemented by the cluster Profiler R package, in which gene length bias was corrected [51]. GO terms, grouped as biological processes, molecular functions, and cellular components, were then assigned to the DEGs to produce an overview of the group of genes present in the transcriptome. GO terms with a corrected P-value less than 0.005 were considered to be significantly enriched in the differentially expressed genes. The cluster Profiler R was used to test the statistical enrichment of differentially expressed genes in KEGG pathways [52].

### Construction of gene coexpression networks and selection of candidate hub genes

WGCNA was performed according to Langfelder and Horvath [15]. WGCNA, an R package for the construction of weighted gene coexpression networks is freely available at https://horvath.genetics.ucla.edu/html/CoexpressionNetwork/Rpackages/WGCNA/. The filtered data were used to construct the network and identify modules. The network analysis was performed by Cytohubba plugin based on Cytoscape and the high degree genes which play a critical role in the protein-protein network (PPI), were identified as hub genes in each module according to the intramodular connectivity KME and correlation with module eigengenes [53].

### Quantitative reverse transcription (RT)-PCR and semiquantitative RT-PCR

RNA isolation and qRT-PCR were performed as previously reported [54, 55]. Relative gene expression levels were calculated using the 2^–ΔΔCT^ method [56]. The obtained data represented three biological replicates, with two technical replicates each. Semiquantitative RT-PCR was performed as described in Chen et al. [57] using specific primers. The obtained data represented three biological replicates. The 18S ribosomal RNA (rRNA) was used as the internal control [23]. All the primers used in this study are shown in Additional file 1: Table S9.

## Additional files

Additional file 1: Supporting tables. **Table S1.** Summary of the sequencing data of Chinese cordyceps transcriptome at different growth stages. **Table S2.** Shared differentially expressed genes among all the five comparisons between adjacent growth stages. **Table S3.** GO functional classification of differentially expressed genes. **Table S4.** KEGG enriched pathway analysis of differentially expressed genes. **Table S5.** KEGG enriched pathway analysis of genes in blue, turpuoise, yellow, purple and magenta modules. **Table S6.** Transcription factor in blue, turpuoise, yellow, purple and magenta modules and FPKM analysis. **Table S7.** Sexual development-related genes functionally verified in *Aspergillus nidulans* and *Neurospora crassa*. **Table S8.** A list of homologous of MAPK-cascaded components and related proteins of *Aspergillus nidulans* and *Neurospora crassa*. **Table S9.** Oligonucleotide primer sequences used in this study. (XLSX 325 kb)

Additional file 2: **Figure S1.** Pearson correlation between samples. **Figure S2.** The distribution of genome-wide gene transcription levels derived from the RNA-seq data. **Figure S3.** Correlation between module membership and gene significance in each module. **Figure S4.** Protein-protein interaction (PPI) network of genes in the blue (A), turquoise (B), yellow (C), purple (D) and magenta (E) modules. **Figure S5.** Semi-quantitative RT-PCR analysis of mating genes in six stages. **Figure S6.** Mating genes expression analysis in different stages by semi-quantitative RT-PCR. **Figure S7.** Putative signal transduction pathways regulating fruiting body development in *O. sinensis*. (DOCX 3075 kb)

## Abbreviations

DEG: differentially expressed genes
DF: developed fruiting body
FPKM: Fragments Per Kilobase of exon model per Million mapped fragments
GO: Gene Ontology
GS: gene significance
HMG: high mobility group
HY: hyphae
KEGG: Kyoto Encyclopedia of Genes and Genomes
KME: eigengene connectivity
MAT: Mating type
MF: mature fruiting body
MM: module membership
MS: module significance
PR: primordium
RT-qPCR: Quantitative reverse transcription - Polymerase Chain Reaction
ST: sclerotium
TF: transcription factor
WGCNA: Weighted gene coexpression network analysis
YF: young fruiting body
