# Supplementary material for: Developmental transcriptomics of Chinese cordyceps reveals gene regulatory network and expression profiles of sexual development-related genes
Source: BMC Genomics. 2019 May 4;20:337. doi: 10.1186/s12864-019-5708-z (PMC6500587; doi:10.1186/s12864-019-5708-z)

**Developmental transcriptomics of Chinese cordyceps reveals gene regulatory network and expression profile of sexual development-related genes**

Xiao Li^a, b^, Fen Wang^a^, Qing Liu^a, b^, Quanping Li^c^, Zhengming Qian^c^, Xiaoling Zhang^a^, Kuan Li^a^, Wenjia Li^c†^, Caihong Dong^a*†^

^a^State Key Laboratory of Mycology, Institute of Microbiology, Chinese Academy of Sciences, Beijing, China; ^b^University of Chinese Academy of Sciences, Beijing 100039, China; ^c^Key Laboratory of State Administration of Traditional Chinese Medicine, Sunshine Lake Pharma Co., LTD, Dongguan, Guangdong 523850, China; ^†^Equal contributors


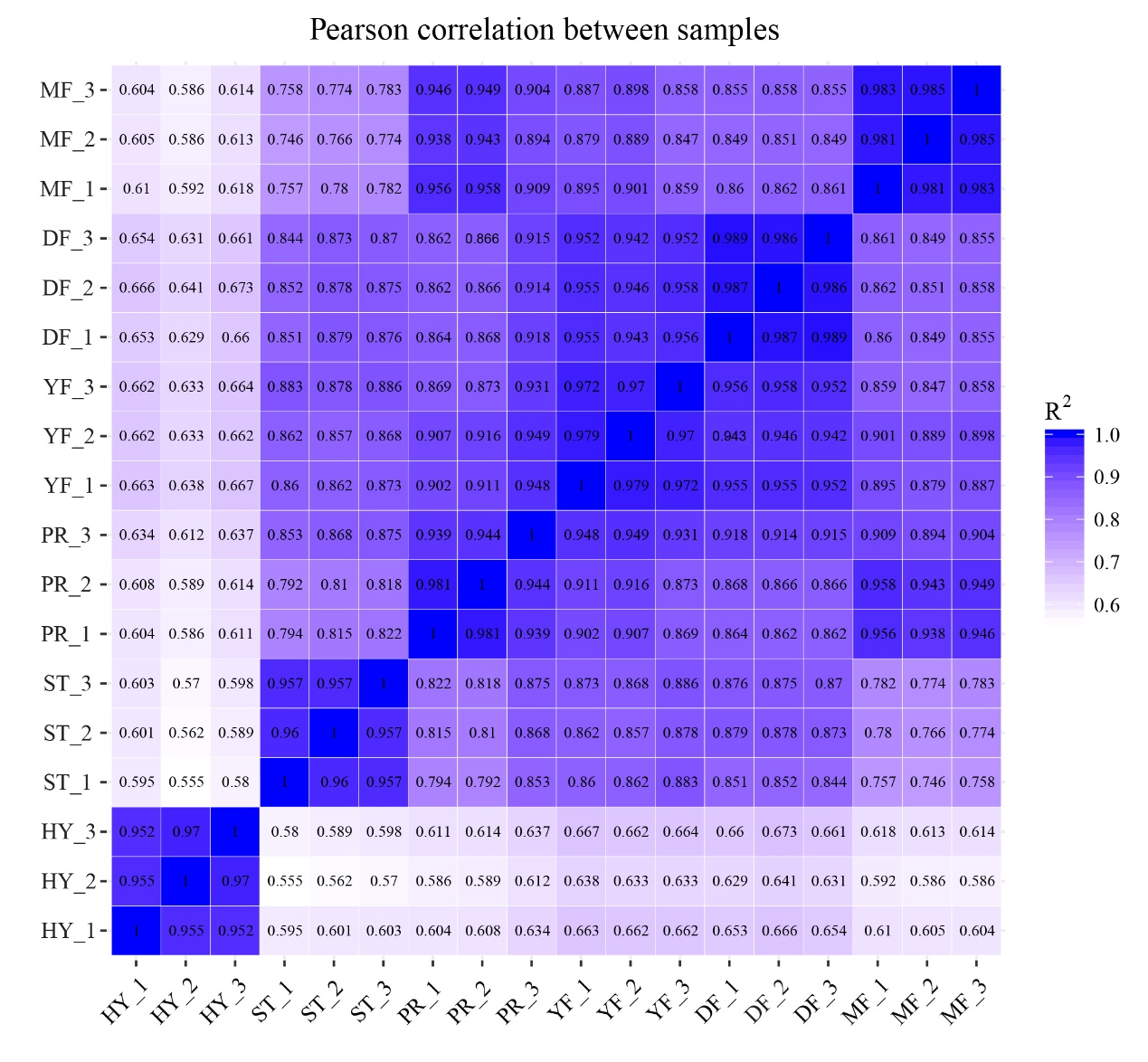
Fig. S1 Pearson correlation between samples.

Fig. S2 The distribution of genome-wide gene transcription levels derived from the RNA-seq data.

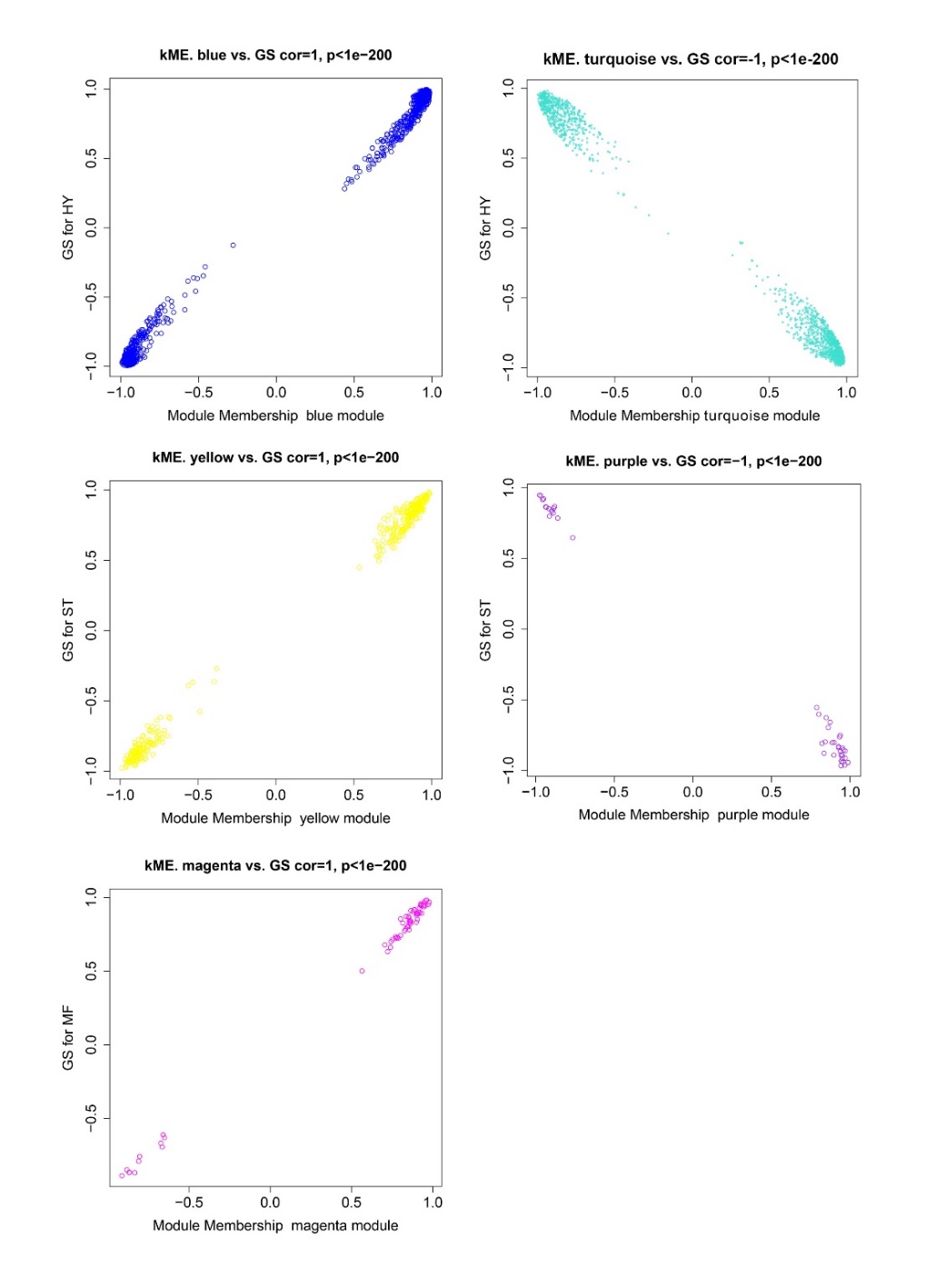
Fig. S3 Correlation between module membership and gene significance in each module.

Fig. S4 Protein-protein interaction (PPI) network of genes in the blue (A), turquoise (B), yellow (C), purple (D) and magenta (E) modules.


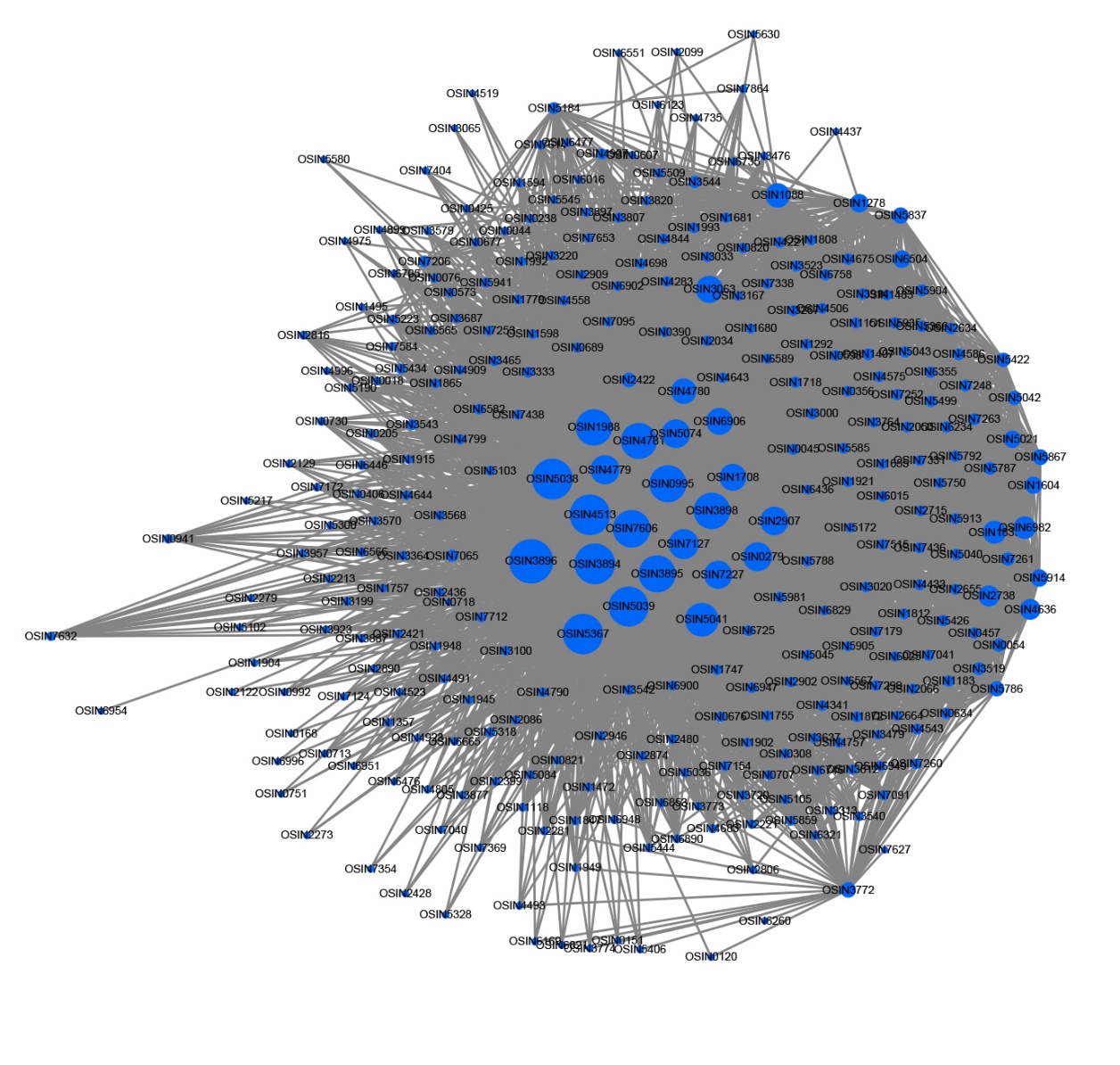
A blue


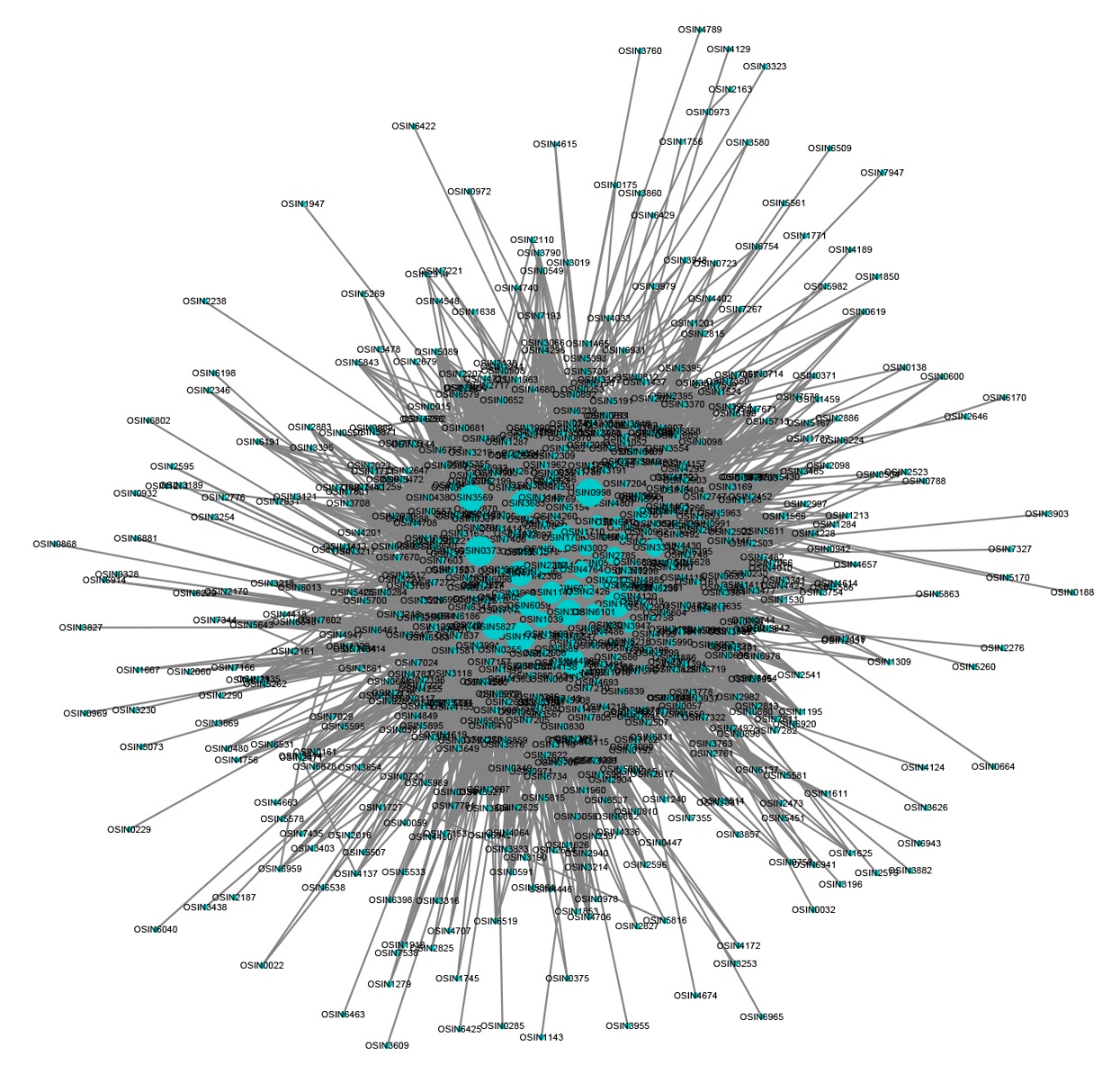
B turquoise


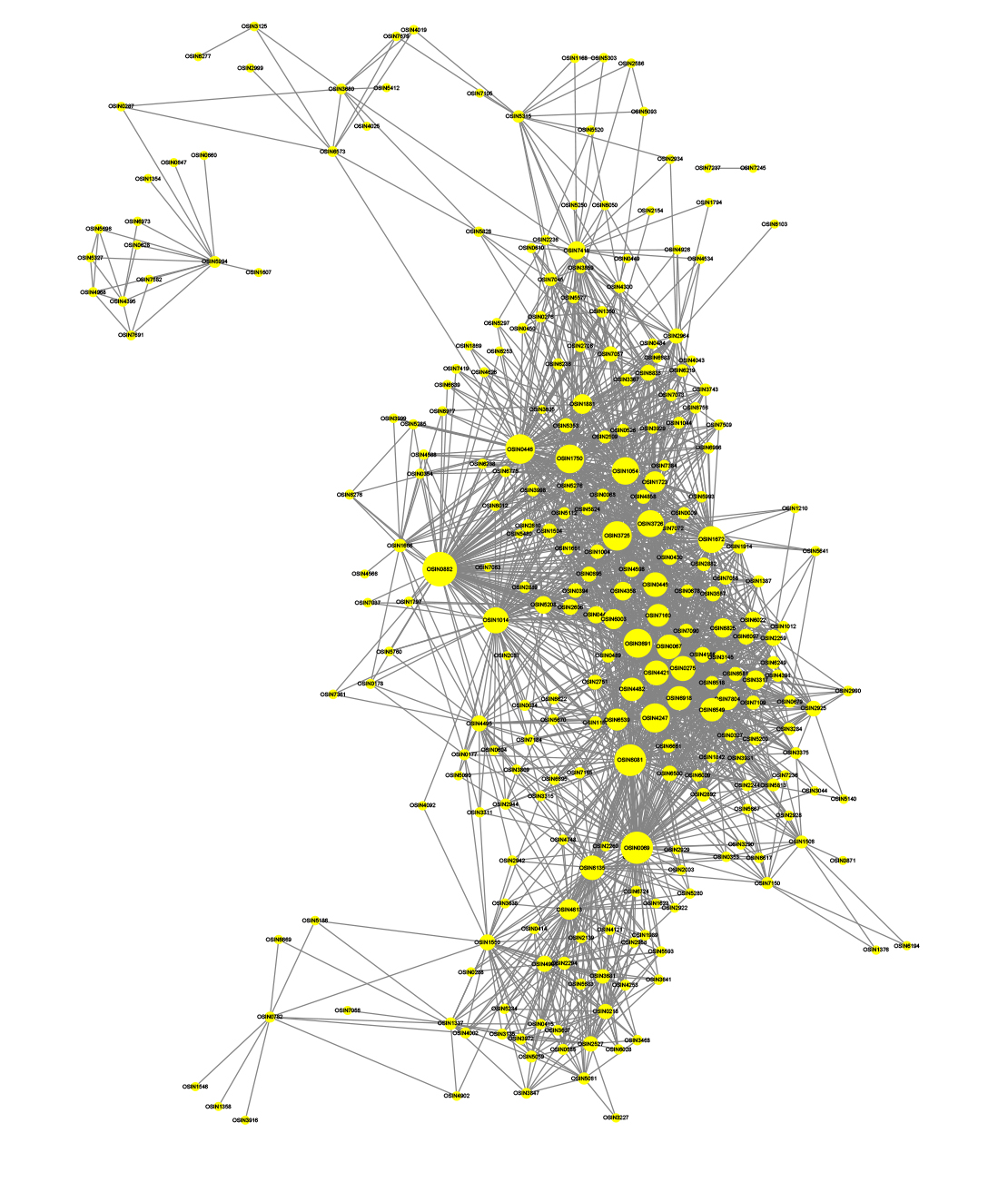
C yellow


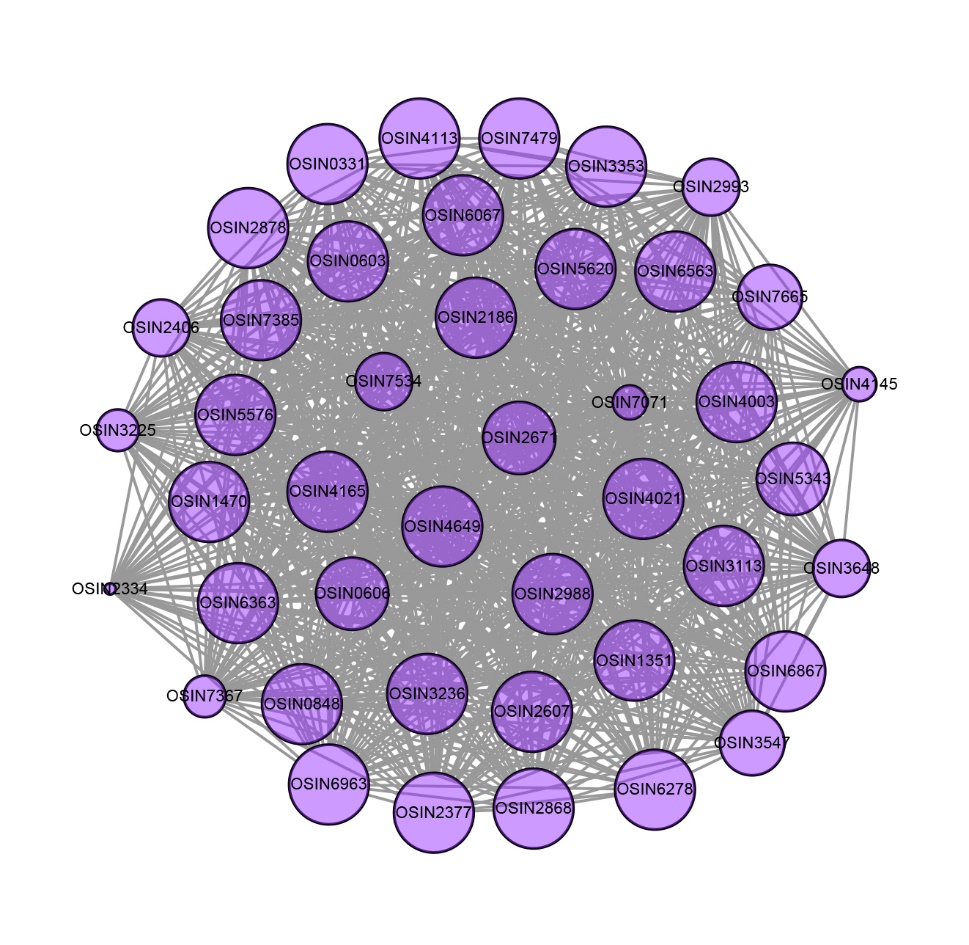
D purple


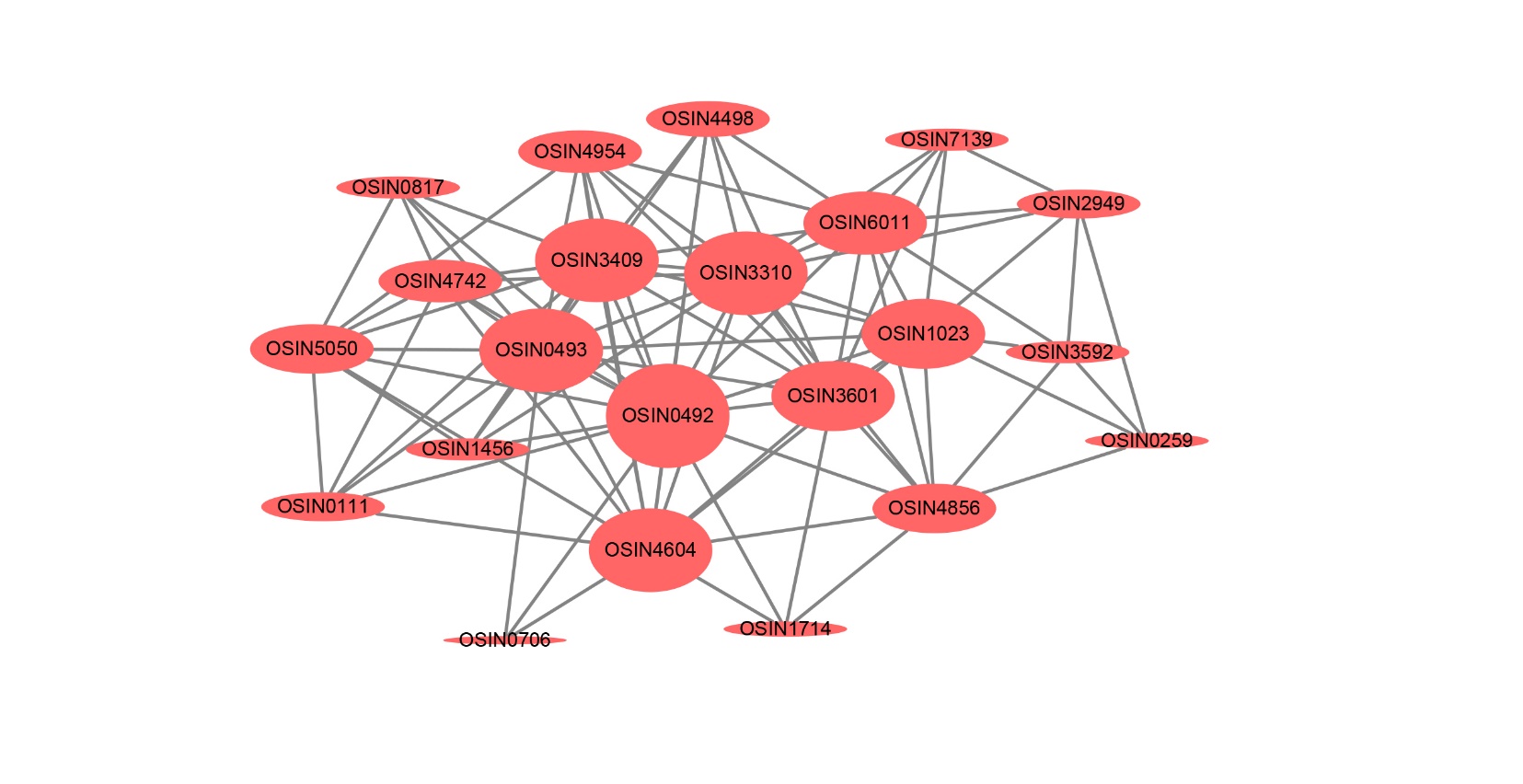
E magenta

The network was constructed using Cytoscape 3.4 software.


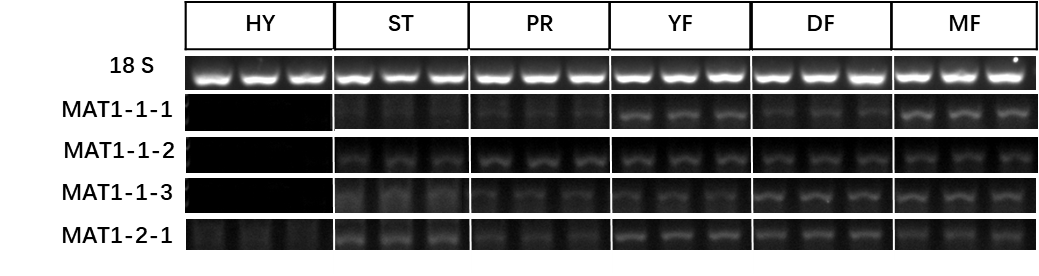
Fig. S5 Semi-quantitative RT-PCR analysis of mating genes in six stages.


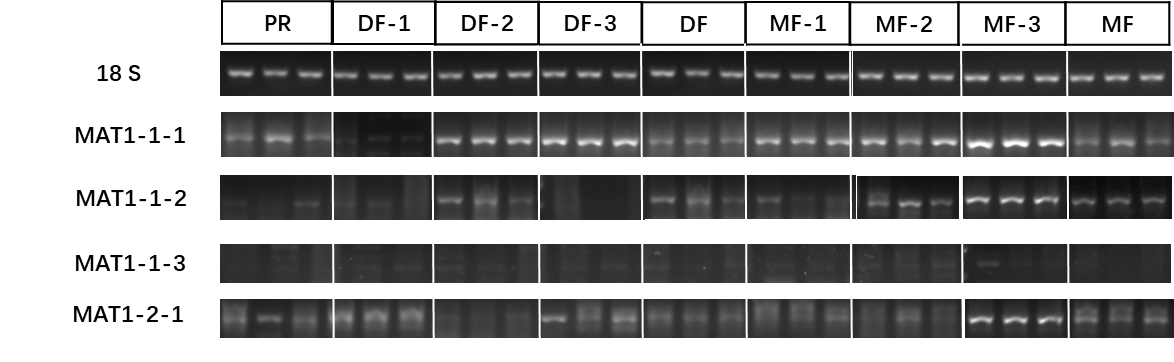
Fig. S6 Mating genes expression analysis in different tissues of Chinese cordyceps by semi-quantitative RT-PCR

Fig. S7 Putative signal transduction pathways regulating fruiting body development in *O. sinensis*. The dashed lines show that the PKA pathway is not involved in regulating fruiting.


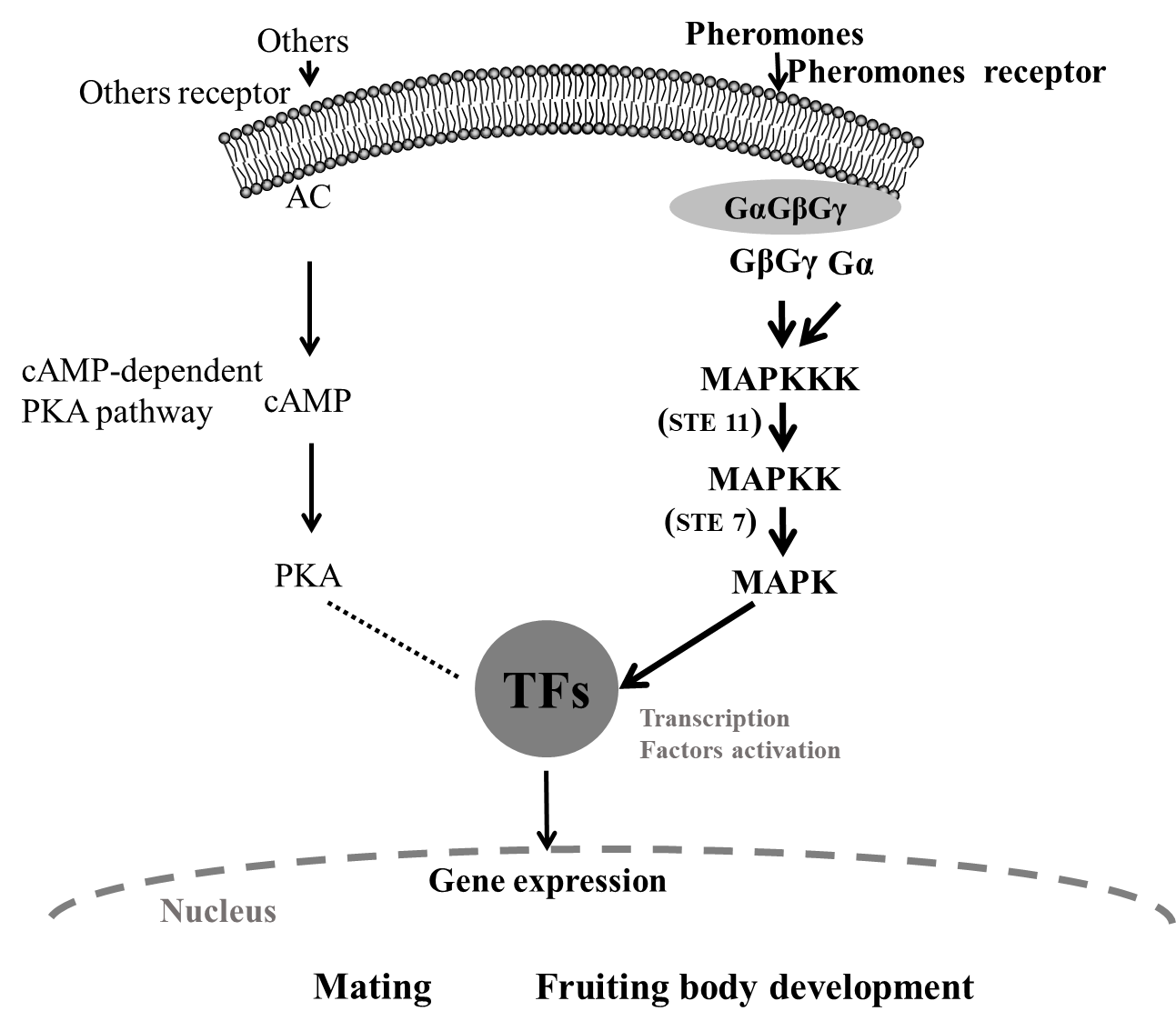

Supplement: Supplementary file 2 — Figure S1. Pearson correlation between samples. Figure S2. The distribution of genome-wide gene transcription levels derived from the RNA-seq data. Figure S3. Correlation between module membership and gene significance in each module. Figure S4. Protein-protein interaction (PPI) network of genes in the blue (A), turquoise (B), yellow (C), purple (D) and magenta (E) modules. Figure S5. Semi-quantitative RT-PCR analysis of mating genes in six stages. Figure S6. Mating genes expression analysis in different stages by semi-quantitative RT-PCR. Figure S7. Putative signal transduction pathways regulating fruiting body development in O. sinensis. (DOCX 3075 kb) [file 12864_2019_5708_MOESM2_ESM.docx]
